# Accelerated strain construction and characterization of *C. glutamicum* protein secretion by laboratory automation

**DOI:** 10.1007/s00253-022-12017-7

**Published:** 2022-06-27

**Authors:** Carolin Müller, Patrick J. Bakkes, Patrick Lenz, Vera Waffenschmidt, Laura M. Helleckes, Karl-Erich Jaeger, Wolfgang Wiechert, Andreas Knapp, Roland Freudl, Marco Oldiges

**Affiliations:** 1 Institute of Bio- and Geosciences IBG-1, Biotechnology, Forschungszentrum Jülich GmbH, 52425 Jülich, Germany; 2 Institute of Biotechnology, RWTH Aachen University, 52062 Aachen, Germany; 3 Institute of Molecular Enzyme Technology, Heinrich Heine University Düsseldorf, Forschungszentrum Jülich, 52425 Jülich, Germany; 4 Computational Systems Biotechnology (AVT.CSB), RWTH Aachen University, 52062 Aachen, Germany; 5 Present Address: Castrol Germany GmbH, 41179 Mönchengladbach, Germany

**Keywords:** Laboratory automation, Sec secretion, Screening, Signal peptide, Ribosome binding site, Split GFP assay

## Abstract

**Abstract:**

Secretion of bacterial proteins into the culture medium simplifies downstream processing by avoiding cell disruption for target protein purification. However, a suitable signal peptide for efficient secretion needs to be identified, and currently, there are no tools available to predict optimal combinations of signal peptides and target proteins. The selection of such a combination is influenced by several factors, including protein biosynthesis efficiency and cultivation conditions, which both can have a significant impact on secretion performance. As a result, a large number of combinations must be tested. Therefore, we have developed automated workflows allowing for targeted strain construction and secretion screening using two platforms. Key advantages of this experimental setup include lowered hands-on time and increased throughput. In this study, the automated workflows were established for the heterologous production of *Fusarium solani* f. sp. *pisi* cutinase in *Corynebacterium glutamicum*. The target protein was monitored in culture supernatants via enzymatic activity and split GFP assay. Varying spacer lengths between the Shine-Dalgarno sequence and the start codon of *Bacillus subtilis* signal peptides were tested. Consistent with previous work on the secretory cutinase production in *B. subtilis*, a ribosome binding site with extended spacer length to up to 12 nt, which likely slows down translation initiation, does not necessarily lead to poorer cutinase secretion by *C.* *glutamicum*. The best performing signal peptides for cutinase secretion with a standard spacer length were identified in a signal peptide screening. Additional insights into the secretion process were gained by monitoring secretion stress using the *C. glutamicum* K9 biosensor strain.

**Key points:**

*• Automated workflows for strain construction and screening of protein secretion*

*• Comparison of spacer, signal peptide, and host combinations for cutinase secretion*

*• Signal peptide screening for secretion by C. glutamicum using the split GFP assay*

**Supplementary Information:**

The online version contains supplementary material available at 10.1007/s00253-022-12017-7.

## Introduction

Intracellular high-level production of target proteins in bacteria can lead to protein aggregation in inclusion bodies. Moreover, the reducing cytoplasmic environment typically hinders the proper folding of proteins containing disulfide bonds making secretion of such proteins to the periplasm or the extracellular space a desirable step (Georgiou and Valax 1996; Villaverde and Carrio 2003). Secretion of a target protein into the extracellular space allows purification directly from the culture medium, avoiding a cell disruption step and thus simplifying downstream processing. Efficient secretory protein production requires a suitable secretion host, such as the prominent Gram-positive model strain *Bacillus subtilis*. Its ability to secrete proteins in the gram-per-liter range makes it an interesting host for industrial production, e.g., for detergent enzymes (Schallmey et al. 2004; van Dijl and Hecker 2013). However, *B. subtilis* secretes various endogenous extracellular proteases that might degrade the target protein, thereby reducing production yields (Wu et al. 1991). Although protease-reduced strains exist (Murashima et al. 2002; Nguyen et al. 2011; Wu et al. 1991), other hosts represent promising alternatives, among them the diderm Gram-positive *Corynebacterium glutamicum*, which exhibits an intrinsically low extracellular proteolytic activity (Vertès 2013). It is long time used in the industry for the large-scale production of amino acids such as l-glutamate and l-lysine (Hermann 2003; Leuchtenberger et al. 2005), as well as for the production of other small molecules like l-ornithine as summarized by Liu et al. (2021). Recently however, *C.* *glutamicum* also became of interest for recombinant protein production and signal peptide-guided protein secretion via the Sec and Tat pathway (Freudl 2017; Lee and Kim 2018).

A bottleneck in all hosts used for Sec-dependent protein secretion is to find a suitable combination of a signal peptide and the heterologous target protein. The secretion efficiency cannot be predicted so far and is also influenced by cultivation conditions, among other factors (Hemmerich et al. 2019a). Instead, signal peptide libraries are systematically screened (Brockmeier et al. 2006a; Fu et al. 2018; Watanabe et al. 2009). In addition to homologous signal peptides, it has been shown that heterologous signal peptides can also enable protein secretion in closely related strains (Degering et al. 2010) and even in strains of different genera, e.g., *B.* *subtilis* signal peptides with *C. glutamicum* as secretion host (Hemmerich et al. 2016). Further optimization of the secretion performance can be done by semi-targeted or untargeted approaches such as site saturation (Caspers et al. 2010) or random mutagenesis (Bakkes et al. 2021) of the signal peptide sequence. In addition to the signal peptide, the 5’ untranslated region and in particular the ribosome binding site spacer between Shine-Dalgarno sequence and start codon impact translation initiation and thus also secretion performance (Sauer et al. 2018; Volkenborn et al. 2020). All these approaches require the testing of numerous different variants. A secretion stress biosensor was designed for *C.* *glutamicum* in which the stress-responsive *htrA* gene, which codes for the periplasmic housekeeping protease HtrA, was replaced by an *eyfp* gene. The fluorescence response of this *C.* *glutamicum* K9 biosensor strain was shown to be dose-dependent on the secretion of target proteins and it is a valuable indicator for secretion associated stress (Bakkes et al. 2021; Jurischka et al. 2020).

Screening of different strain variants in a short time can also be enabled by automated workflows, miniaturization, and parallelization (Hemmerich et al. 2018). Target proteins can directly be detected in supernatant samples via activity measurements, provided that the target protein has enzymatic activity and that assays suitable for automation are established. A convenient alternative detection method is the activity-independent split GFP assay. For this purpose, the C- or N-terminus of the target protein is covalently linked to the 11th β-sheet of a superfolder GFP (GFP11-tag). After addition of the non-fluorescent GFP1-10 consisting of the remaining β-sheets, the holo-GFP can self-assemble and the chromophore is formed (Cabantous et al. 2005). Due to the small size of the GFP11-tag, secretion performance is not affected by the tag and holo-GFP fluorescence can be correlated to the amount of target protein in the supernatant as demonstrated for the cutinase enzyme (Bakkes et al. 2021; Knapp et al. 2017; Müller et al. 2021). In addition, the split GFP assay measurements can be combined with activity measurements for data normalization (Santos-Aberturas et al. 2015). The split GFP assay can easily be automated and the cultivation and screening workflow is adaptable to other target proteins with accessible GFP11-tag, even if these proteins do not possess enzymatic activity or when an established automatable activity-based assay is lacking (Dörr et al. 2016; Müller et al. 2021).

In this work, a set of 14 different *B.* *subtilis* signal peptides was tested for secretion of the *Fusarium solani* f. sp. *pisi* cutinase by *C. glutamicum*. Plasmid-based strain generation was accelerated using a low-cost liquid handling system with open-source-based software. Protein secretion by these strains was tested in an automatic screening with a more complex robotic platform. The cutinase activity and the split GFP assay were used to detect the target protein in the culture supernatants and the best performing signal peptides were identified by both detection methods. In addition to the different signal peptides, also the impact of 9 ribosome binding site spacer lengths ranging from 4 to 12 nt was investigated. The results were compared to results obtained for the secretion host *B.* *subtilis* (Volkenborn et al. 2020) and we showed that an extended spacer length to up to 12 nt does not necessarily lead to poorer cutinase secretion by *C.* *glutamicum*. Selected combinations were additionally tested in the *C.* *glutamicum* K9 strain to assess the stress associated with the secretory production of the different constructs.

## Materials and methods

### Strains, media, and growth conditions

Plasmids used in this study are listed in Table 1. For pCMEx-based cutinase secretion by *C.* *glutamicum*, strains ATCC 13032 (Kinoshita et al. 1957) and ATCC 13032 K9 (Jurischka et al. 2020) were transformed by electroporation (van der Rest et al. 1999). Brain Heart Infusion (BHI, Carl Roth, Germany), BHI supplemented with 91 g/L d-sorbitol (BHIS), or CGXII medium (Keilhauer et al. 1993) with 20 g/L glucose was used for cultivation at 30 °C, each supplemented with 30 µg/mL kanamycin unless stated otherwise.

**Table 1:** Plasmids used in this work

Plasmid	Description	Reference
pET22b-sfGFP1-10	pET22b( +) with *sfgfp1-10* gene under control of P_T7_, Amp^R^	Knapp et al. (2017)
pBS-4nt-SPPel-Cut11	pBSMul1-based *E. coli/B.* *subtilis* shuttle vector with 4 nt spacer length and *Nde*I/*Xba*I inserted fragment containing Pel signal peptide sequence, *F.* *solani* f. sp. *pisi cut1* gene, and C-terminal GFP11-tag sequence, Km^R^	Volkenborn et al. (2020)
pBS-Xnt-SPNprE-Cut11	pBSMul1-based *E. coli/B.* *subtilis* shuttle vector with X nt spacer length (X = 4–12) and *Nde*I/*Xba*I inserted fragment containing NprE signal peptide sequence, *F.* *solani* f. sp. *pisi cut1* gene, and C-terminal GFP11-tag sequence, Km^R^	This study
pBSMul-SPMix-SPNprE-YoaJ-GFP11	pBSMul3-based *E. coli/B.* *subtilis* shuttle vector with 5 nt spacer length, *Hin*dIII/*Eco*RI inserted fragment containing NprE signal peptide sequence and *Eco*RI/*Xba*I inserted fragment containing *B. subtilis yoaJ* gene and GFP11-tag sequence, Km^R^	K. Volkenborn, unpublished
pUC57-Insert-Amp	pUC57 with insert consisting of P_tac_, 12 nt spacer with sequence [a]_9_cat, *B.* *subtilis* signal peptide sequence from *yncM*, *Actinia equina blue chromoprotein aeCP597* under the control of the constitutive P_em7_ and GFP11-tag sequence, Amp^R^	Synthesized (Synbio Technologies, USA)
pUC57-Cutinase-Amp	pUC57 with *F.* *solani* f. sp. *pisi cut1* gene, Amp^R^	Synthesized (Synbio Technologies, USA)
pPBEx2	*E. coli*/*C.* *glutamicum* shuttle vector, Km^R^	Bakkes et al. (2020)
pCMEx12	pPBEx2*-*based plasmid with *Nde*I restriction site removed by blunting, new insert consisting of P_tac_, 12 nt spacer with sequence [a]_9_cat, *B.* *subtilis* signal peptide sequence from *yncM*, *Actinia equina blue chromoprotein aeCP597* under the control of the constitutive P_em7_ and GFP11-tag sequence via circular polymerase extension cloning, Km^R^	This study
pCMEx[4–11]	pCMEx12-based *E. coli*/*C.* *glutamicum* shuttle vector with [4–11] nt spacer length with sequence [a]_1-9_cat cloned into pCMEx12 via* Pst*I/*Nde*I	This study
pCMEx[4–12]-[SP]	pCMEx[4–12] with *B. subtilis* signal peptide sequence from *yncM* exchanged with other *B. subtilis* signal peptide sequences via* Nde*I and *Eco*RI	This study
pCMEx[4–12]-[SP]-Cutinase	pCMEx[4–12]-SP with P_em7_-*aeCP597* exchanged with *F.* *solani* f. sp. *pisi cut1* by Golden Gate assembly using BsaI. *Cut1* gene in frame with N-terminal *B.* *subtilis* signal peptide and C-terminal GFP11-tag sequence, P_tac_, [4–12] nt spacer length	This study

*B. subtilis* TEB1030 (Eggert et al. 2003) was grown at 37 °C in shaking flasks filled to 1/10 with lysogeny broth (LB) with Miller’s modifications (Miller 1972) containing 50 µg/mL kanamycin for maintenance of Cut11-encoding pBSMul1 (Brockmeier et al. 2006b) derivatives. Transformation was carried out using naturally competent *B.* *subtilis* cells (Anagnostopoulos and Spizizen 1961).

*Escherichia coli* was cultivated in LB with Miller’s modifications (Miller 1972) at 37 °C. Strains DH5α and TOP10 (both Thermo Fisher Scientific, USA) were used for DNA cloning. LB was supplemented with 50 µg/mL kanamycin or 100 µg/mL ampicillin for strains containing pCMEx-based plasmids or pBSMul1- and pBSMul3-based plasmids, respectively. Detector protein GFP1-10 for the split GFP assay was produced with *E.* *coli* BL21(DE3) pET22b-sfGFP1-10 (Knapp et al. 2017) in LB supplemented with 100 µg/mL ampicillin. All solid media were prepared by addition of 15 g/L agar–agar.

### Optical density measurements

Optical density was measured in a UV-1800 spectrophotometer (Shimadzu, Japan) at 580 nm (OD_580_, *E. coli* and *B.* *subtilis*) or 600 nm (OD_600_, *C.* *glutamicum*). 0.9% (w/v) NaCl was used as a blank and for appropriate sample dilution.

### Production and purification of GFP1-10 for split GFP assay

Liquid cultures of *E. coli* BL21(DE3) pET22b-sfGFP1-10 were incubated at 37 °C, 180 rpm, and 25 mm shaking diameter in baffled flasks filled to 1/10 volume with LB. A single colony from LB agar was used to inoculate a pre-culture of 10 mL LB. After incubation for 16 h, 50 mL or 100 mL LB were inoculated from the pre-culture to an OD_580_ of 0.05. Intracellular GFP1-10 production was induced by adding IPTG to a final concentration of 200 µM after 2 h of incubation. Cells were harvested after 5 more hours by centrifugation (9283 × *g*, 4 °C, 5 min) and cell pellets were stored at −20 °C until further processing. Cell disruption and GFP1-10 purification were done as described elsewhere (Müller et al. 2021). Briefly, cell pellets containing GFP1-10 were resuspended to an OD_580_ of 12 in TNG buffer containing 100 mM Tris–HCl pH 7.4, 100 mM NaCl, and 10% (w/v) glycerol. Cells were disrupted by French press with four iterations at 15,000 psi. The inclusion body fraction containing GFP1-10 was isolated and purified by three repeats of centrifugation (9283 × *g*, 4 °C, 5 min) and resuspension in 15 mL TNG buffer. After an additional centrifugation, 1 mL 9 M urea per 75 mg pellet containing the inclusion bodies was added for unfolding of GFP1-10 and samples were centrifuged (14,000 × *g*, 4 °C, 10 min). Each 400 µL supernatant containing soluble GFP1-10 was transferred and mixed with 10 mL TNG buffer for protein refolding. This GFP1-10 solution was stored at −20 °C and additional 10 mL TNG buffer with 10 mM EDTA was added directly before use in split GFP assays with samples from secretion with *B.* *subtilis* and *C.* *glutamicum* K9. For split GFP assays with samples from secretion by *C.* *glutamicum* ATCC 13032, the EDTA was omitted.

### General molecular biology techniques

If not further specified, standard molecular cloning techniques were used (Green and Sambrook 2012). Restriction enzymes, Q5^®^ Hot Start High-Fidelity DNA Polymerase, and T4 DNA ligase were obtained from New England Biolabs (USA) and used according to manufacturer’s instructions. The DNA was manually purified using the NucleoSpin Plasmid (NoLid) or NucleoSpin Gel and PCR Clean‑up together with the NucleoVac 24 Vacuum Manifold (all from Macherey–Nagel, Germany) and the DNA concentration was measured in a NanoDrop ND-1000 UV–Vis spectrophotometer (Thermo Fisher Scientific, USA). DNA fragments were analyzed by gel electrophoresis or microchip electrophoresis (MCE™-202 MultiNA, Shimadzu, Japan) according to the manufacturer’s instructions, using the 1 kb Plus DNA Ladder (New England Biolabs, USA) for size comparison in all cases. DNA sequencing and oligonucleotides were purchased from Eurofins (Germany) and DNA synthesis was performed by Synbio Technologies (USA). For cloning of plasmids for *B.* *subtilis*, restriction enzymes and the Phusion^®^ DNA Polymerase from Thermo Fisher Scientific were used according to manufacturer’s instructions. DNA samples were sequenced by LGC Genomics (Germany). All oligonucleotide sequences are provided in the Supplementary Table S1.

### Construction of cutinase secretion plasmids

For construction of the plasmid pCMEx12, the *Nde*I restriction site of pPBEx2 (Bakkes et al. 2020) was removed by blunting with DNA Polymerase I, Large (Klenow) Fragment (New England Biolabs, USA), according to manufacturer’s instructions. To allow for easy replacement of the ribosome binding site spacer, the *B.* *subtilis* signal peptide, and the target gene with a GFP11-tag sequence as described below, a suitable DNA fragment was synthesized by Synbio Technologies (USA) and delivered in the form of the plasmid pUC75-Insert-Amp. This DNA fragment also contains the gene of a reporter protein under the control of a constitutive promoter that stains *E. coli* colonies blue. The pUC75-Insert-Amp and the pPBEx2 with the removed *Nde*I restriction site were separately linearized and amplified by the Q5^®^ Hot Start High-Fidelity DNA Polymerase (New England Biolabs, USA) with forward and reverse primers CPEC-Backbone and CPEC-Insert, respectively (Supplementary Table S1). Resulting DNA fragments were purified by gel extraction and fused by circular polymerase extension cloning. A reaction mix with 1 × Q5 Reaction Buffer, Q5^®^ Hot Start High-Fidelity DNA Polymerase (both New England Biolabs, USA), 200 µM dNTPs, 500 ng backbone, and insert in an insert-to-vector molar ratio of 1:2 was prepared. Thermocycling conditions were 30 s at 98 °C for initial denaturation, 5 cycles of 10 s at 98 °C, 30 s at 68 °C, and 128 s at 72 °C, followed by a final extension at 72 °C for 2 min. The complete sequence of pCMEx12 is deposited under the GenBank accession number OM801558 and a plasmid map is provided in the Supplementary Fig. S1a.

The exchange of the spacer sequence was done by cassette mutagenesis (Wells et al. 1985). 5 µM of two partial complementary oligonucleotides (Supplementary Table S1) was mixed in 1 × T4 ligase buffer (New England Biolabs, USA). Annealing was done in a thermocycler by heating to 90 °C for 5 min, 56.2 °C for 20 min, reducing the previous temperature by 5 °C, holding for 120 min, and slowly cooling to room temperature. The resulting double stranded fragments have short overhangs for directional cloning and were ligated with pCMEx12 that was digested with *Pst*I and *Nde*I and purified by gel extraction.

The plasmids pCMEx[4–12] were used for exchange of the signal peptide sequence using the same approach as for the exchange of the spacer sequence, with the notable differences that the pCMEx[4–12] were digested with *Nde*I and *Eco*RI and that the temperature profile for annealing of oligonucleotides was adapted to 85 °C in the second step.

The cutinase gene without its native signal peptide sequence from pUC57-Cutinase-Amp (Synbio Technologies, USA) was inserted into plasmids pCMEx[4–12]-[SP] by Golden Gate assembly with the Type IIS restriction enzyme BsaI according to manufacturer’s instructions. Both plasmids were provided in the reaction mix with an equimolar ratio and the temperature profile for assembly was 30 repetitions of alternating 37 °C for 1 min and 16 °C for 5 min, followed by 10 min at 37 °C and heat-inactivation of enzymes at 80 °C for 20 min. The exemplary sequence of pCMEx8-NprE-Cutinase is deposited under the GenBank accession number OL456171. Since the constitutively expressed reporter gene is exchanged with the cutinase gene during Golden Gate assembly, the *E. coli* colonies are no longer blue after transformation (Supplementary Fig. S1b). Plasmids were purified from three white colonies per construct. As an additional control, restriction digestion was performed with *Not*I-HF and *Eco*RI-HF that would cut twice with the inserted cutinase gene and only once without, and DNA fragments were analyzed by capillary electrophoresis. The workflow from the starting plasmid pCMEx12 to the secretion screening is depicted in Supplementary Fig. S2.

For construction of the pBS-Xnt-SPNprE-Cut11 plasmid series, *cut11* gene was isolated from plasmid pBS-4nt-SP_Pel_-Cut11 (Volkenborn et al. 2020) by hydrolysis with *Eco*RI and *Xba*I, prior to ligation into the likewise hydrolyzed plasmid pBSMul-SPMix-SPNprE-YoaJ-GFP11 (K. Volkenborn, unpublished) which led to a plasmid with an in frame fusion of the NprE signal sequence and the *cut11* gene. Thereafter, this fragment was amplified with the primer pair fw-NdeI-SPNprE and rev-GFP11-XbaI (Supplementary Table S1) introducing an *Nde*I site at the 5’-end and an *Xba*I site at the 3’-end. The PCR product was then hydrolyzed with *Nde*I and *Xba*I and ligated into the likewise hydrolyzed pBS-Xnt-GUS11 plasmid series (Lenz et al. 2021) to construct the vector series pBS-Xnt-SP_NprE_-Cut11 with spacer lengths of 4–12 nt for cutinase-GFP11 secretion facilitated by the NprE signal peptide.

### Automation of plasmid construction

Automated protocols for accelerated strain construction were developed for the OT-2 robot with multichannel pipettes P20 GEN2, P300, and P300 GEN2, as well as the Thermocycler and the Magnetic Module (Opentrons, USA). Protocols written for the OT-2 Python Protocol API Version 2 for Golden Gate assembly, *E. coli* heat-shock transformation, restriction digestion, and plasmid purification using the Wizard^®^ MagneSil^®^ Plasmid Purification System (Promega, USA) are provided in the JuBiotech Git repository (Müller and Helleckes 2022).

### Cutinase secretion by* B. subtilis*

For *B. subtilis* cutinase expression cultures, a 10 mL overnight culture was inoculated with a single transformant and grown at 37 °C under aerobic conditions. This culture was used to inoculate 10 mL LB medium to an OD_580_ of 0.05, prior to cultivation at 37 °C, 130 rpm, and 50 mm shaking diameter for 6 h. The *cut11* gene was expressed under control of the strong constitutive P_HpaII_ promoter (Guan et al. 2015).

### Monitoring cutinase secretion using the *C. glutamicum* ATCC 13032 K9 biosensor strain

Cutinase secretion experiments were performed using *C. glutamicum* ATCC 13032 K9 biosensor cells that enable the monitoring of recombinant protein secretion by means of cellular fluorescence (Jurischka et al. 2020). All biosensor cell cultivations were performed in a FlowerPlate using a BioLector^®^ I (Beckman Coulter, USA) at 85% relative humidity, 1200 rpm, and 30 °C. To avoid evaporation, the FlowerPlate was covered with a sterile gas permeable membrane. Bacterial growth was monitored online by measuring the backscatter at 620 nm, while the eYFP fluorescence (λ_ex_ 508 nm, λ_em_ 532 nm) of the biosensor cells was measured in parallel. The cell-specific biosensor fluorescence output in a.u. at the end of the cultivation was calculated as the eYFP fluorescence per backscatter signal.

The cutinase secretion experiments conducted with the biosensor cells carrying the different recombinant plasmids were performed essentially as described by Bakkes et al. (2021) with media supplemented with 25 µg/mL kanamycin to maintain the plasmids. In brief, the biosensor cells were first cultivated in BHIS medium for 5–6 h. Hereafter, 50 µL of the BHIS cultures was transferred to a new FlowerPlate containing 800 µL CGXII medium with 10 g/L glucose in each well and growth was continued overnight. Next, the OD_600_ of the cultures was determined and appropriate amounts were transferred to a new FlowerPlate containing fresh medium in each well, yielding a starting OD_600_ of ~1 (total volume of 800 µL per well). Cultivation was then continued in the BioLector^®^ and 4 h after inoculation, IPTG was added (250 µM final concentration), and cultivation was sustained for another 20 h. The amount and activity of the cutinase in the culture supernatants at the end of the cultivation were determined by manual split GFP assay and cutinase activity measurements.

### Manual cutinase activity assay

Cutinase activity in the culture supernatants was determined by a spectrophotometric assay using 4-nitrophenyl palmitate (4NPP) as a substrate analogue (Winkler and Stuckmann 1979), essentially as described previously (Bakkes et al. 2021; Volkenborn et al. 2020). Cell-free culture supernatants of the recombinant strains were appropriately diluted in 66.5 mM Sørensen’s phosphate buffer pH 8.0 and 20 µL of diluted *C. glutamicum* supernatants or 10 µL diluted *B.* *subtilis* supernatants were transferred to a standard 96-well microtiter plate (MTP). Assay reagent was prepared freshly by mixing 1 volume of 30 mg 4NPP dissolved in 10 mL isopropanol with 9 volumes of Sørensen’s buffer pH 8.0, containing 1.11 mg/mL gum arabic and 2.3 mg/mL sodium deoxycholate. The enzymatic reaction was initiated by the addition of assay reagent to a final volume of 200 µL in each MTP well. The MTP was then transferred to a plate reader (Infinite M1000 Pro, Tecan, Switzerland, for *C. glutamicum* or SpectraMax 250, Molecular devices, Germany, for *B. subtilis* supernatant samples) preheated to 37 °C. After brief and vigorous mixing, the absorbance at 410 nm was measured at 37 °C for 15 min with 1 min or 30 s intervals with supernatant samples from *C.* *glutamicum* or *B.* *subtilis*, respectively. The cutinase activity was calculated from the linear slope using a molar extinction coefficient of 15,000 M^−1^ cm^−1^. For each cultivated recombinant strain, at least two independent clones were tested, and activity measurements were performed in duplicates for secretion by *C.* *glutamicum* or in biological and technical triplicates for secretion experiments using *B. subtilis*.

### Manual split GFP assay

The split GFP assay was carried out essentially as described previously (Knapp et al. 2017). To reconstitute holo-GFP, 20 µL of undiluted culture supernatant containing the GFP11-tagged cutinase were mixed with 180 µL of detector GFP1-10 solution in each well of a black flat-bottom MTP. The MTP was covered to avoid evaporation and incubated at 20 °C under gentle agitation to enable assembly of holo-GFP and chromophore maturation. For *C.* *glutamicum* supernatant samples, the MTP was covered with a sterile gas permeable membrane and incubated for 24 h. For *B.* *subtilis* supernatant samples, the MTP was covered with the MTP lid and incubated for 16 h before fluorescence measurement. In all cases, chromophore excitation was performed in an Infinite M1000 Pro plate reader (Tecan, Switzerland) at 485 nm (bandwidth 10 nm), while the fluorescence emission was measured from 505 to 550 nm in 5 nm steps and using an appropriate gain factor. The GFP-specific emission maximum at 510 nm was used for subsequent calculations. Replicates were measured as described for manual cutinase activity assay.

### Automated cultivation and screening workflow for *C. glutamicum* ATCC 13032

The automated cutinase-GFP11 secretion screening workflow as described by Müller et al. (2021) was extended by automated pre-culture handling. A microbioreactor (BioLector^®^ Pro, Beckman Coulter, USA) integrated into a robotic platform (Freedom EVO^®^ 200, Tecan, Switzerland, schematic in Supplementary Fig. S3) was used for cultivation at 30 °C, 1400 rpm, and ≥ 85% relative humidity with online measurement of backscatter and dissolved oxygen. BioLector^®^ Pro data was parsed during the experiment using the bletl package (Osthege et al. 2022a, 2022b). During the experiment, worklists are written with robotools (Osthege et al. 2021) based on backscatter measurements that can be loaded and executed by the robotic platform using a device control system developed by M. Osthege and J. Hemmerich (Forschungszentrum Jülich GmbH, in preparation). Twelve cryo cultures in vials as well as CGXII medium in a trough were placed on deck of the robotic platform. For pre-cultures, 780 µL CGXII were transferred to 12 wells of a 48-well FlowerPlate with optodes (MTP-48-BOH 1) covered with sealing foil for automation (both Beckman Coulter, USA). 20 µl of each cryo culture was used to inoculate one of the pre-culture wells. As soon as a pre-culture in the exponential phase exceeded a certain device-dependent backscatter threshold, three new wells for main cultures were autonomously filled with 780 µL CGXII and inoculated with 20 µL of the respective pre-culture. The main cultures were induced in the early exponential phase by adding IPTG to a final concentration of 200 µM once they exceeded a certain backscatter threshold. The cells were harvested (3756 × *g*, 4 °C, 6 min) 4 h after induction and the supernatants were stored in a deep well plate on a cooling carrier. After all cultivations in the current run were completed, cutinase-GFP11 was detected in the supernatant by automated activity and split GFP assay.

Automated cutinase activity assay with 4NPP as substrate analogue (Winkler and Stuckmann 1979) was conducted as previously described (Hemmerich et al. 2019b). For the reaction mix, one part 3 g/L 4NPP in isopropanol was added to 9 parts 50 mM potassium phosphate buffer pH 8 (KP_i_ buffer) with 2.3 g/L sodium deoxycholate and 1.1 g/L gum arabic. A total of 200 µL were transferred to wells of a MTP and pre-heated to 37 °C. Supernatant was 160 × diluted with KP_i_ buffer and 40 µL was added in analytical duplicates. 4-nitrophenol formation was measured by absorption at 410 nm in 25 s intervals for 40 min at 37 °C in a MTP reader (Infinite^®^ M Nano, Tecan, Switzerland). 40 µl 4-nitrophenol standards in KP_i_ buffer covering a range from 0 to 2 mM were mixed with 200 µL reaction mix and absorption was measured in triplicates for calibration.

For the automated split GFP assay, 20 µL undiluted supernatant was mixed with 180 µL GFP1-10 detector solution in a black MTP with clear bottom as described elsewhere (Knapp et al. 2017). Fluorescence was measured at 20 °C for at least 14 h with 485 nm excitation and 535 nm emission wavelength in a MTP reader (Infinite^®^ M Nano, Tecan, Switzerland) with shaking (1000 rpm, linear mode) between measurements.

### Protein precipitation and SDS-PAGE analysis

The proteins in the supernatant were precipitated using trichloroacetic acid (TCA) as described by Bakkes et al. (2020). In brief, 500 µL supernatant containing cutinase-GFP11 from a BioLector^®^ cultivation was mixed with 60 µL 100% (w/v) TCA and incubated at 4 °C and 600 rpm for at least 1 h. Precipitated proteins were pelleted by centrifugation (20,000 × *g*, 4 °C, 30 min) and resuspended in 100 µL 1 × sample buffer (prepared from Laemmli 2 × Concentrate, Sigma-Aldrich, USA). The pH was adjusted by adding 12 µL 1 M Tris solution and the samples were boiled at 99 °C for 5 min. 10 µl was analyzed on a 4–12% Criterion™ XT Bis–Tris Protein Gel (Bio-Rad Laboratories, USA). 7 µl PageRuler™ Prestained Protein Ladder (10 to 180 kDa, Thermo Fisher Scientific, USA) were added to the gel for molecular weight assessment. Proteins were separated at 200 V with NuPAGE™ MES SDS Running Buffer (Thermo Fisher Scientific, USA). The gel was stained with SimplyBlue™ SafeStain (Thermo Fischer Scientific, USA) according to the manufacturer’s instructions.

## Results

### Strain construction and automation

In this study, various combinations of *B.* *subtilis* signal peptides and ribosome binding site spacer sequences ranging from 4 to 12 nt (nucleotide sequence [a]_1-9_cat) between the Shine-Dalgarno sequence and the start codon were tested for secretion of *F.* *solani* f. sp. *pisi* cutinase in *C.* *glutamicum*. For accelerated plasmid construction by automation, traditional cloning cannot be used. Here, the DNA fragments are usually purified by gel extraction between restriction digestion and ligation, which is difficult to automate. Golden Gate assembly as an alternative uses type IIS restriction enzymes such as BsaI that cut outside their recognition site. Since the target plasmid no longer contains the recognition sequence, it is not further digested, unlike other plasmids in the reaction mix. Thus, restriction and ligation can be performed in one-pot setup without gel extraction in between (Engler et al. 2008).

To create a suitable plasmid backbone for Golden Gate assembly, the *Nde*I restriction site in plasmid pPBEx2 (Bakkes et al. 2020) was removed and the insert of the synthetic pUC57-Insert-Amp was integrated via circular polymerase extension cloning. This insert has an altered sequence between the tac promoter and the terminator that consists of a 12 nt ribosome binding site spacer, *B.* *subtilis yncM* signal peptide sequence, *A.* *equina blue chromoprotein aeCP597* under the control of the constitutive EM7 promoter, and a GFP11-tag sequence that is not in frame with *aeCP597*. The resulting plasmid pCMEx12 (GenBank accession number OM801558, plasmid map in Supplementary Fig. S1a) allows for the exchange of the spacer and signal peptide sequence by cassette mutagenesis with restriction enzymes *Pst*I/*Nde*I and *Nde*I/*Eco*RI, respectively. The constitutively expressed reporter gene *aeCP597* can be exchanged with the gene of interest by Golden Gate assembly. After a successful exchange, *E. coli* colonies have lost their blue color and the cutinase gene without its native signal peptide sequence is cloned in frame with the *B.* *subtilis* signal peptide sequence and GFP11-tag sequence under the control of the tac promoter (Supplementary Fig. S1b). The workflow of the sequence exchanges from the starting plasmid pCMEx12 to the secretion screening is shown in the Supplementary Fig. S2.

The exchange of the gene of interest was automated using the Opentrons OT-2 robot with a Thermocycler and Magnetic Module. Unit operations were the Golden Gate assembly, heat-shock transformation of *E. coli*, magnetic beads-based plasmid purification, and restriction digestion as a control for successful Golden Gate assembly in addition to the colony color. Restriction enzymes would cut once before and twice after successful integration of the target gene by Golden Gate assembly and DNA fragments were subsequently analyzed by capillary electrophoresis. If only one DNA fragment was visible, either new transformants were picked for plasmid purification or the Golden Gate assembly was repeated. Python scripts can be found online in the JuBiotech Git repository (Müller and Helleckes 2022).

**Fig. 1:**
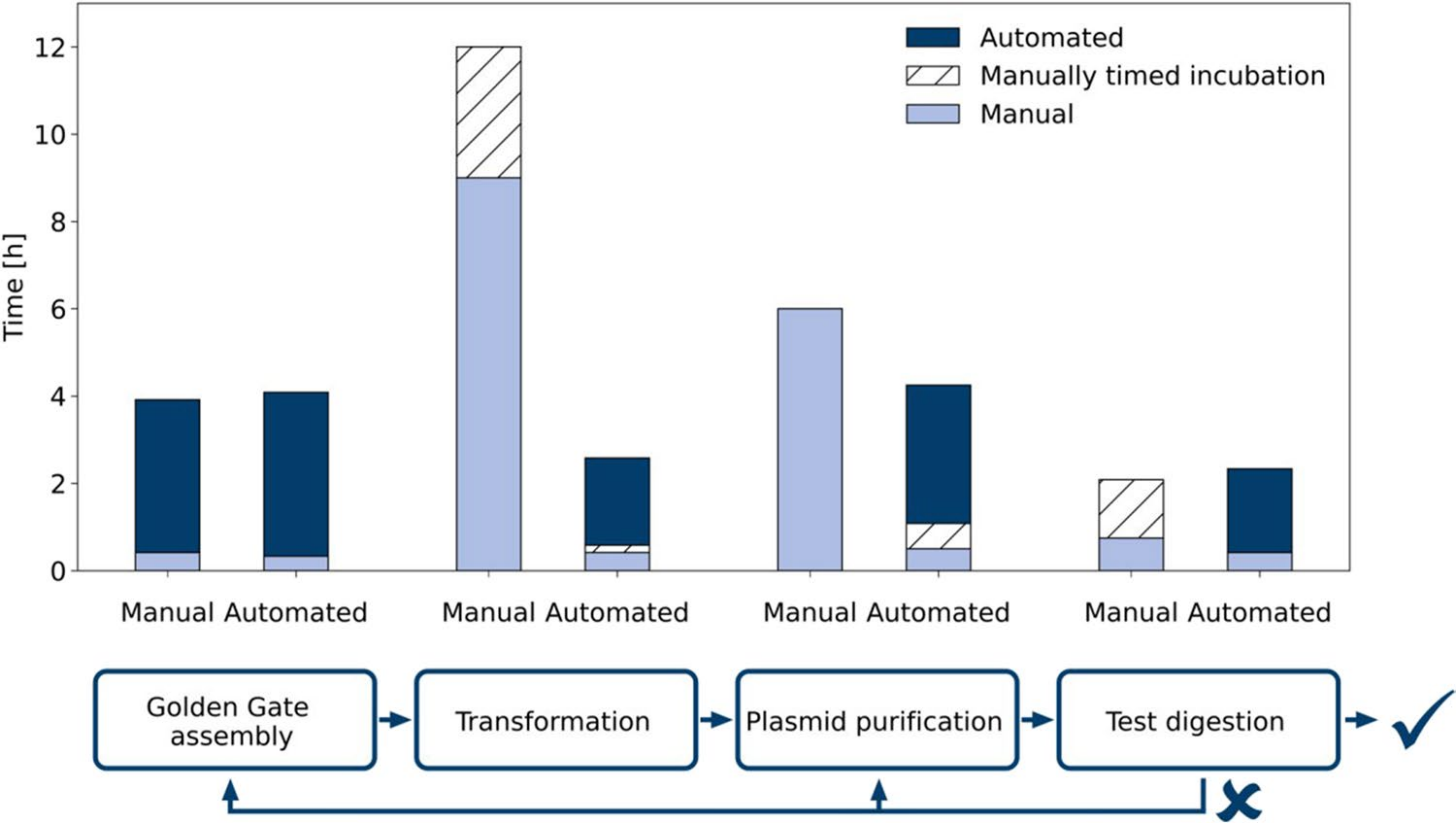
Comparison of automated and manual construction of 96 plasmids. The plasmid construction can be separated into four unit operations: (1) Golden Gate assembly of plasmid carrying the gene of interest and a backbone with the desired spacer and signal peptide combination, (2) heat-shock transformation of *E. coli* with Golden Gate assembly samples, (3) magnetic beads-based plasmid purification from *E. coli*, and (4) restriction digestion of the purified plasmids for additional control. Manually timed incubation implies that the experimenter can do other things during the incubation, but has to do something manually after a given time and thus has to monitor the time. For Golden Gate assembly, either the plasmids with 96 different inserts or backbones were already appropriately diluted in a 96-well plate. 96 samples were handled in parallel in automated plasmid construction workflows. For manual transformation, usually up to 12 samples can be handled in parallel and a second batch can start when the first batch is in the regeneration phase. For plasmid purification, only the steps from cell harvest to plasmid elution were considered and 24 samples at a time were handled manually using the NucleoVac 24 Vacuum Manifold. Analysis of test digestion samples via capillary electrophoresis is not included. More detailed time tables of the unit operations can be found in the Supplementary Tables S2–S5

Comparison of the time required for manual or automated treatment of 96 samples showed an overall reduction of process time to about 58% of the time needed for the manual process (Fig. 1). However, the time savings varied among unit operations. For Golden Gate assembly, both options took about the same time since they do not differ in the automatically running thermocycler protocol. Automated test digestion even took slightly longer, albeit the manual work was reduced compared to manual test digestion. In contrast, automation of heat-shock transformation reduced the overall time to about 75% with only 25 min of manual work left to prepare the robot. The manually laborious plasmid purification by hand took about 6 h, assuming that only 24 samples can be handled in parallel using the NucleoVac 24 Vacuum Manifold. Thus, automation slightly reduced the total time to 4.25 h, but importantly the majority of this process is fully automated and does not require operator supervision.

### Automated screening workflow for cutinase secretion

**Fig. 2:**
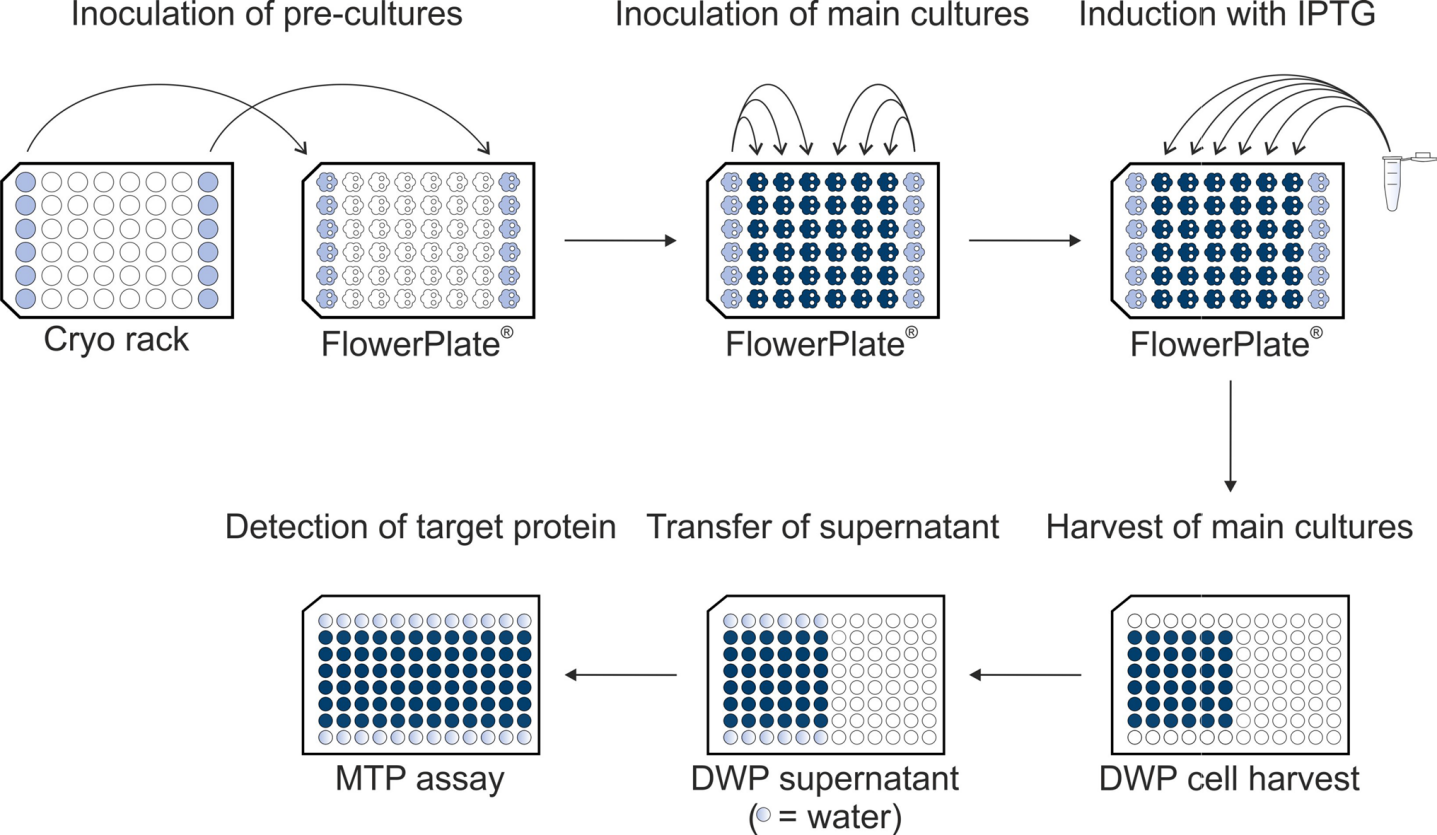
Schematic workflow for automated cultivation and screening. Pre-culture wells in the outer columns of the cultivation plate in a microbioreactor are automatically filled with CGXII medium from a cooled trough on deck of the robotic system and inoculated from thawed cryo cultures in vials. As soon as a pre-culture exceeds a device-dependent backscatter threshold in the exponential phase, three adjacent wells in the same row were filled with medium for the main culture and inoculated from the respective pre-culture. Triggered by the backscatter signal, main cultures are induced in the early exponential phase with IPTG and harvested in a deepwell plate (DWP) after 4 h of target protein production. Cultivation supernatant is stored on deck in a cooling carrier until all main cultures are harvested before detection of target protein in the supernatant by split GFP and activity assay using microtiter plates (MTPs)

**Fig. 3:**
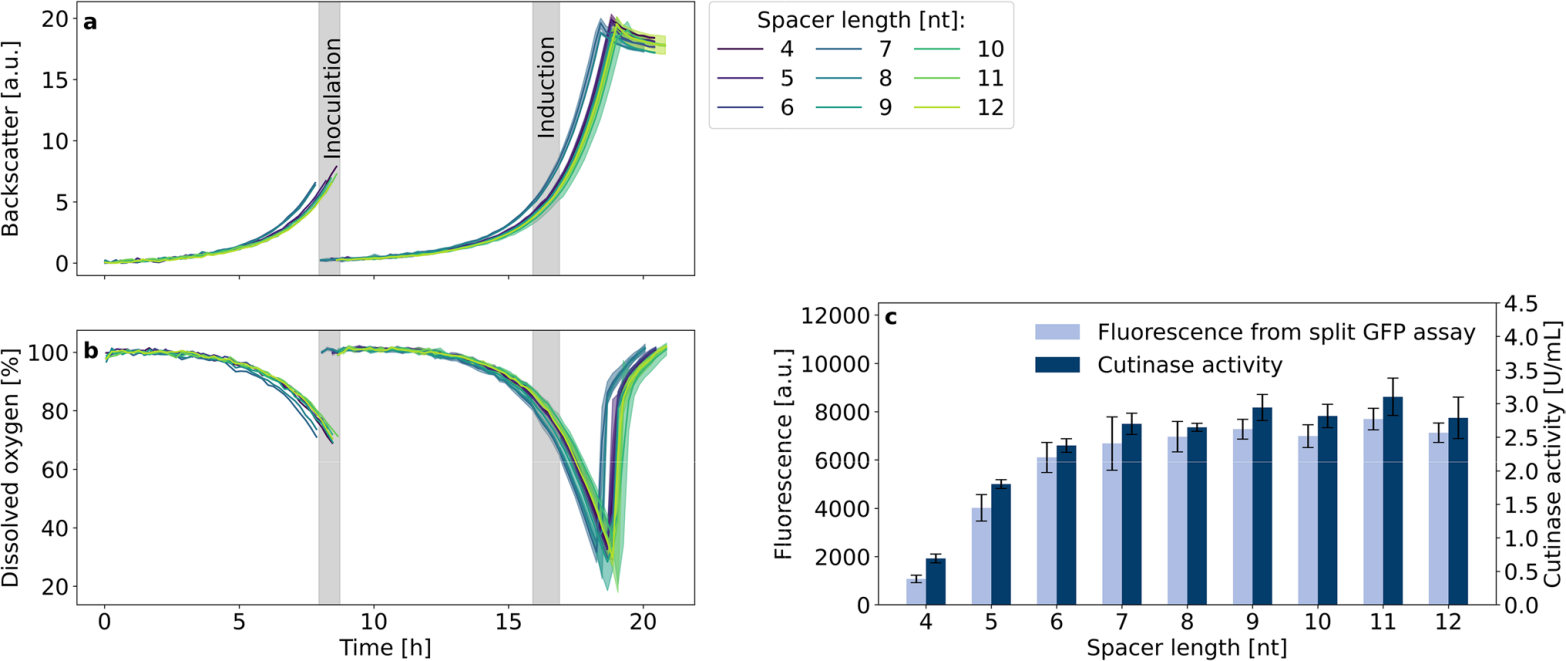
Cutinase-GFP11 secretion with ribosome binding site spacer lengths from 4–12 nt. Backscatter (**a**) and dissolved oxygen (**b**) were measured during cultivation of *C. glutamicum* pCMEx[4–12]-NprE-Cutinase. Pre-cultures inoculated from cryo cultures were used to inoculate three main cultures, which are shown as mean with standard deviation in confidence tubes. Inoculation and induction of main cultures with IPTG to a final concentration of 200 µM were triggered by backscatter signals. Cells were harvested 4 h after induction, and cutinase-GFP11 in the supernatant was detected via the split GFP and cutinase activity assays (**c**) in analytical duplicates of the respective three main cultures. For comparison with the cutinase activity, the maximum holo-GFP fluorescence after saturation was used

Screening of heterologous cutinase secretion was automated using a microcultivation robotic platform with Tecan Freedom EVO^®^ 200, BioLector^®^ Pro, centrifuge, and microplate reader (schematic in Supplementary Fig. S3). The workflow described by Müller et al. (2021) was extended to automated pre-cultures in the BioLector^®^ to omit laborious pre-cultures in flasks and to enable more standardized autonomous handling of pre- and main cultures (Fig. 2). In this workflow, main cultures are performed in triplicates, showing low deviation and a high degree of growth comparability (see Fig. 3a and b). The 12 pre-cultures are inoculated from cryo cultures placed on deck of the robotic system into the wells in the outer columns. Inoculation of three main cultures per pre-culture as well as induction of main cultures with IPTG are triggered individually by the backscatter signal of the cultivation that correlates with the cell density. The three wells next to the pre-culture wells are used for main cultures; i.e., 20 µL of the pre-cultures was transferred from column 1 and 8 to main cultures in column 2–4 and 5–7, respectively. Harvesting is triggered 4 h after induction and cutinase-GFP11 is detected in supernatants after all cultivations are finished by split GFP and cutinase activity assay.

### Impact of the ribosome binding site spacer on the secretion performance

The accelerated plasmid construction workflow followed by the automated robotic cultivation and screening workflow was employed for investigating the impact of the ribosome binding site spacer sequences between the Shine-Dalgarno sequence and the start codon. The pPBEx2 plasmid which is the scaffold for pCMEx-based plasmids contains an 8 nt spacer. In total, 9 spacer sequences ranging from 4 to 12 nt were compared using the *Bacillus subtilis* signal peptide of the protease NprE for cutinase-GFP11 secretion in *C. glutamicum* (Fig. 3).

Although the lag-phases and resulting growth phenotypes of nine different strains varied in the pre-cultures, all main cultures grew with a net cultivation time between 11.6 and 12.3 h from inoculation to harvest in the stationary phase. Such differences in the pre-culture growth were likely to be expected, due to potential deviations in the individual cell viability in the cryo cultures and inoculation density. Such deviation was effectively corrected by the individual inoculation strategy of the main cultures using a backscatter trigger enabling an autonomous inoculation of the main cultures. This led to growth phenotypes with high similarity between the replicates of the 9 individual strains in terms of backscatter biomass signal (Fig. 3a) as well as dissolved oxygen profile (Fig. 3b). The same applied for the secretion performance data, showing good comparability for the replicate data, but with considerably different secretion performance for the different spacer lengths (Fig. 3c). The secretion performance was evaluated based on direct measurement of cutinase activity and fluorescence data from split GFP assay. The assembly of holo-GFP and the formation of chromophores was similar to previous experiments (Müller et al. 2021). Results from the split GFP and cutinase activity assays showed standard deviations in the range of 10% and below, and the corresponding data are in good agreement, ranging from around 1074 to 7124 a.u. and 0.7 to 2.8 U/mL, respectively. The spacer length of 8 nt was already among the top performance values with the highest values for the 11 nt spacer. Strikingly, for 7–12 nt, a considerable plateau region of high secretion performance was observed and no decrease at a spacer length beyond the 8 nt was found. The opposite was observed for smaller spacer lengths, with a clear negative effect of approx. 75% decrease in cutinase activity for a 4 nt ribosome binding site spacer. It seems that the reduction of the spacer length has a more severe effect on secretory production than its extension, which had a minor effect only.

**Fig. 4:**
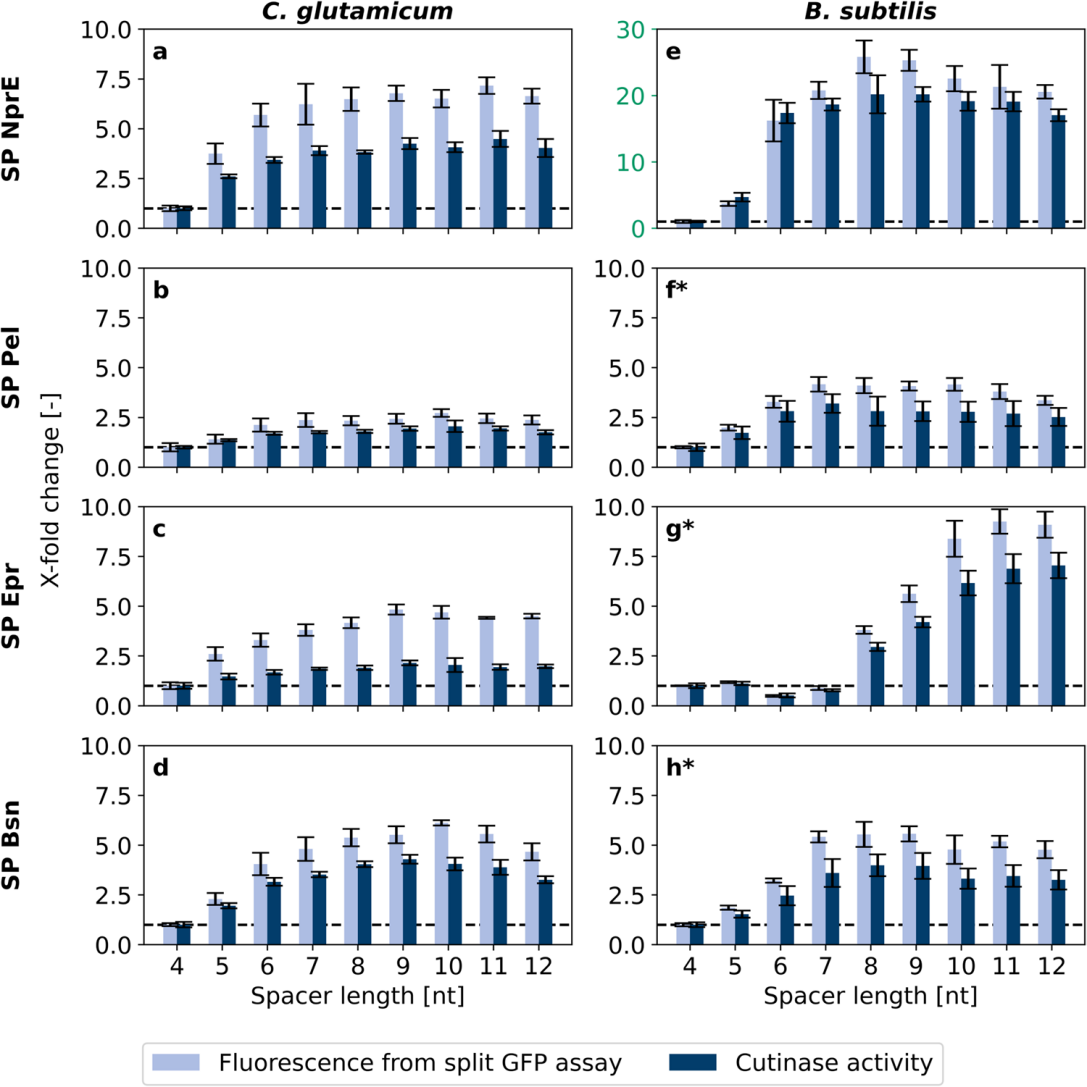
Impact of spacer length on cutinase-GFP11 secretion with *B.* *subtilis* signal peptides NprE, Pel, Epr, and Bsn. *C.* *glutamicum* (**a**–**d**) or *B.* *subtilis* (**e**–**h**) were used as secretion hosts. Cutinase-GFP11 was detected in supernatant samples of *C.* *glutamicum* by cutinase activity and split GFP assay in analytical duplicates of three cultivation supernatants, respectively. Cutinase-GFP11 in *B.* *subtilis* cultivation supernatants was detected in triplicates. Enzymatic activity and holo-GFP fluorescence after saturation were normalized to x-fold changes relative to the respective strain with a 4 nt ribosome binding site spacer (dashed line). The Y-axis scaling for subfigure **e** differs from the others and is highlighted in green. *data from Volkenborn et al. (2020), Creative Commons CC BY license, see Reprints and Permissions

Next, the automated screening of clones harboring plasmids with 4–12 nt spacer lengths was repeated with *B. subtilis* signal peptides Pel, Epr, and Bsn for cutinase-GFP11 secretion in *C. glutamicum*. The growth phenotypes of the respective variants were similar to those of NprE (Supplementary Fig S4–S6). To facilitate comparison, the cutinase activity and split GFP fluorescence data for all signal peptides including NprE were each normalized to x-fold change relative to the strain with the 4 nt spacer, as this strain was expected to show the lowest secretion performance (Fig. 4a–d). Results were compared to those with *B. subtilis* as host (Fig. 4e–h), where data marked with an asterisk are from Volkenborn et al. (2020) (Creative Commons CC BY license, see Reprints and Permissions).

In general, the data for cutinase activity and split GFP assay were comparable for the two strains and the 4–12 nt spacers used provided a clear picture of the effects with slightly higher x-fold changes for the split GFP data. For the four signal peptides in *C. glutamicum*, a very similar response to changes of the spacer length was observed, showing the lowest values for the 4 nt ribosome binding site spacer and highest for 8–10 nt spacers with a gradual increase in between. This seems to confirm the initial results for the NprE signal peptide and may indicate a universal response to spacer length variation.

For *B. subtilis*, the picture seems to be more heterogeneous. Again, a small 4 nt spacer length showed lowest performance and spacer lengths of 7 nt and longer were superior. The overall highest x-fold changes were measured for the signal peptide NprE with an 8 nt ribosome binding site spacer with an increase of about 20-fold in activity and 26-fold in split GFP assay fluorescence compared with the shortest spacer length (Fig. 4e). However, the individual response to nucleotide elongation was different for the four signal peptides. For the signal peptide NprE, a stepwise increase to high activity was observed going from 5 to 6 nt (Fig. 4e). For signal peptides Pel and Bsn, a gradual increase similar as in *C.* *glutamicum* was found (Fig. 4f* and h*). Although a gradual increase was also observed for the Epr signal peptide, this positive effect of spacer elongation required at least 8 nt to show increased formation of cutinase activity (Fig. 4g*). For this signal peptide, a significantly longer spacer led to optimized secretion performance.

Overall, the secretion performance was lowest with the 4 nt spacer and was gradually increased by elongation with one or more nucleotides with 8–10 nt being necessary to obtain close to optimal cutinase activity. Interestingly, secretion performance was much less sensitive to longer ribosome binding site spacers than for those being shorter than 8 nt.

### Signal peptide screening

**Fig. 5:**
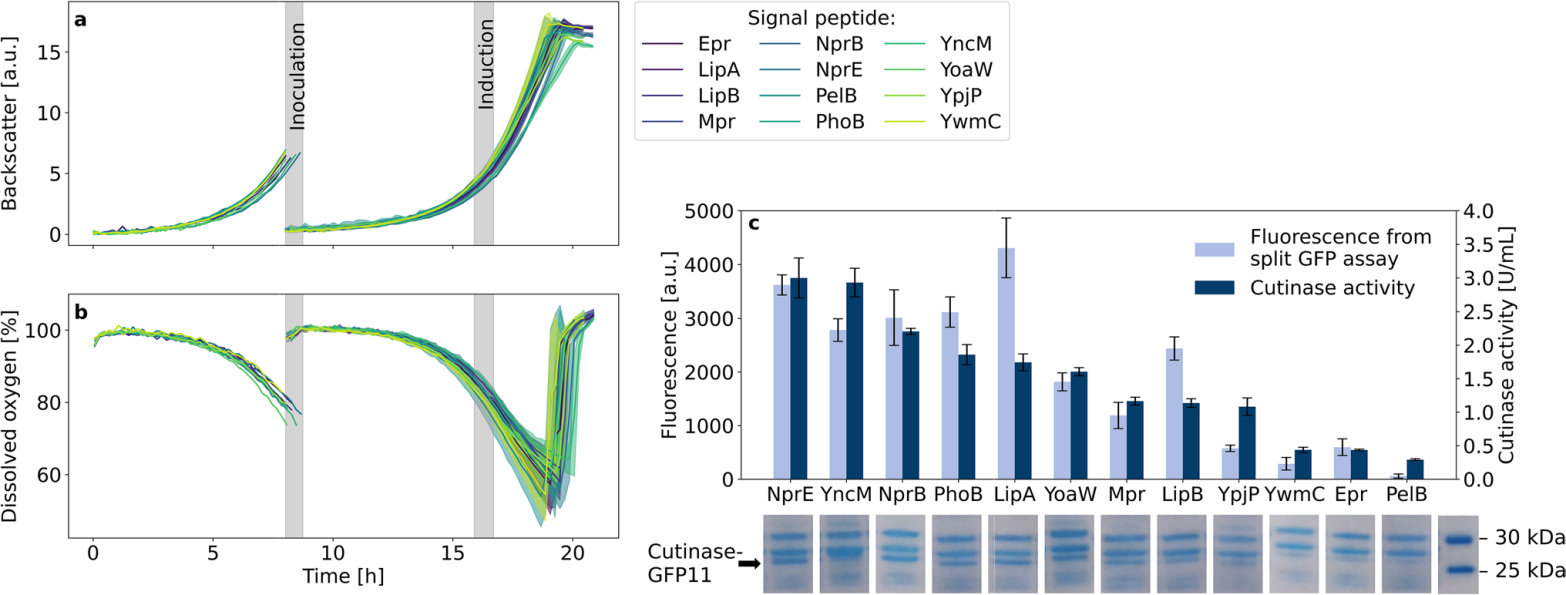
Cutinase-GFP11 secretion with different signal peptides and a 8 nt ribosome binding site spacer. *C.* *glutamicum* pCMEx8-[SP]-Cutinase online cultivation data of backscatter (**a**) and dissolved oxygen (**b**) were measured. Pre-cultures were inoculated from cryo cultures. Triggered by the backscatter signal in the early exponential phase, pre-cultures were used to inoculate three main cultures that are shown as mean with standard deviation in confidence tubes. Induction of main cultures with IPTG to a final concentration of 200 µM was triggered by backscatter signals in the exponential phase and cells were harvested 4 h later. Cutinase-GFP11 in the supernatant was detected via SDS-PAGE, split GFP assay, and cutinase activity measurements (**c**). Assays were conducted in analytical duplicates of the respective three main cultures with determination of fluorescence after saturation of self-assembled GFP. Supernatants of the same strain were pooled and proteins were precipitated with TCA before SDS-PAGE

A *B. subtilis* signal peptide screening for cutinase-GFP11 secretion by *C.* *glutamicum* was performed based on the plasmid pCMEx8 which harbors an 8 nt spacer sequence (Fig. 5). An extended set of signal peptide sequences that were all previously tested for cutinase secretion with *B. subtilis* (Brockmeier et al. 2006b) with high (YncM, Epr), medium (PhoB, LipA, YoaW), or low (Mpr, NprE, YpjP) secretion performance was used. Other signal peptide sequences were already used for cutinase secretion by *C. glutamicum* (NprE, Pel, YwmC, YpjP) as described elsewhere (Hemmerich et al. 2019a, 2016; Jurischka et al. 2020; Müller et al. 2021). Two additional signal peptides have been applied for high secretion of other target proteins, i.e., the signal peptide sequences of PhoB (Zhang et al. 2016) and NprB (Zhang et al. 2005).

Growth phenotypes of these *C.* *glutamicum* strain variants were very similar to those observed in Fig. 3 and Supplementary Fig. S4–S6, exhibiting a duration of the main cultures of 12–12.8 h (Fig. 5a and b). For the signal peptides YncM and YpjP, a more linear than exponential growth was observed with lower final backscatter values. These results may indicate a potential metabolic burden or cellular stress due to cutinase-GFP11 secretion. Cutinase-GFP11 activity in the supernatant showed substantial differences for the extended set of signal peptides used. The highest activities were measured for signal peptides NprE and YncM with approximately 3 U/mL, while only very low activities were detected with signal peptides YwmC, Epr, and PelB. Strikingly, based on the split GFP assay, the highest amount of cutinase-GFP11 was detected with signal peptide LipA. Higher fluorescence than expected based on the activity results was also measured for the signal peptide LipB. It should be noted that the split GFP assay basically determines the amount of cutinase-GFP11 protein and does not directly report on the cutinase activity. This indicates that a lot of inactive protein is secreted with the signal peptide LipA, whereas this does not seem to be the case with the signal peptide NprE, for example. However, the five signal peptides facilitating the highest extracellular cutinase activities, i.e., NprE, YncM, NprB, PhoB, and LipA, were identified as top performers in both assays. In all supernatants, at least small amounts of cutinase-GFP11 could be identified with TCA precipitation followed by SDS-PAGE at the expected molecular weight of 25.1 kDa.

### Secretion stress measured by *C.* *glutamicum* K9

**Fig. 6:**
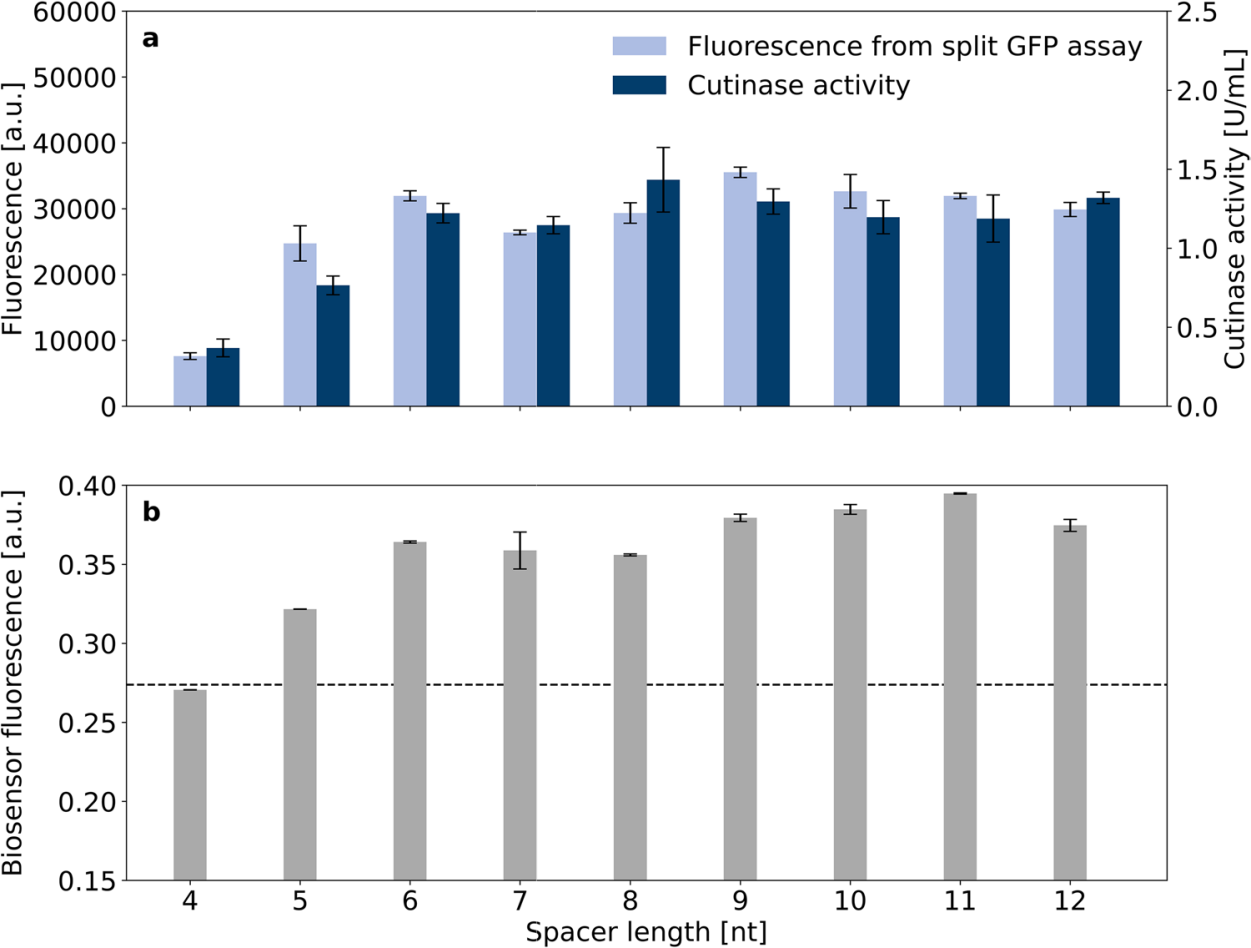
Secretion performance and secretion stress associated with cutinase-GFP11 secretion using the signal peptide NprE in combination with ribosome binding site spacers of different lengths (4–12 nt). Two independent clones of *C.* *glutamicum* K9 pCMEx[4–12]-NprE-Cutinase were cultivated and secreted cutinase-GFP11 was detected via split GFP assay and cutinase activity measurements in analytical duplicates (**a**). At the end of the cultivation, the cell-specific eYFP fluorescence was determined of two independent clones and the average is shown with standard deviation (**b**). The dashed line indicates the normal background fluorescence associated with secretion stress in the absence of cutinase expression

Previous studies on *C. glutamicum* have indicated that high-level production of secretory proteins is typically accompanied by secretion stress at the cellular envelope (Bakkes et al. 2021; Jurischka et al. 2020; Kleine et al. 2017). To assess the impact of the spacer length and signal peptide on the secretion stress associated with cutinase secretion, plasmids were additionally transferred to the *C.* *glutamicum* K9 secretion stress biosensor strain. In this strain, the secretion stress responsive gene *htrA*, which codes for the periplasmic housekeeping protease HtrA, is replaced by an *eyfp* gene that is under control of the *htrA* promoter. The resulting biosensor strain enables the monitoring of the Sec-dependent protein secretion stress by means of intracellular eYFP fluorescence, which would not be possible with the *C. glutamicum* ATCC 13032 production strain (Jurischka et al. 2020). In a proof of concept, *C.* *glutamicum* K9 pCMEx[4–12]-NprE-Cutinase were cultivated with online eYFP measurement, while cutinase-GFP11 in the supernatant at the end of the cultivation was detected manually with the split GFP and cutinase activity assays (Fig. 6).

**Fig. 7:**
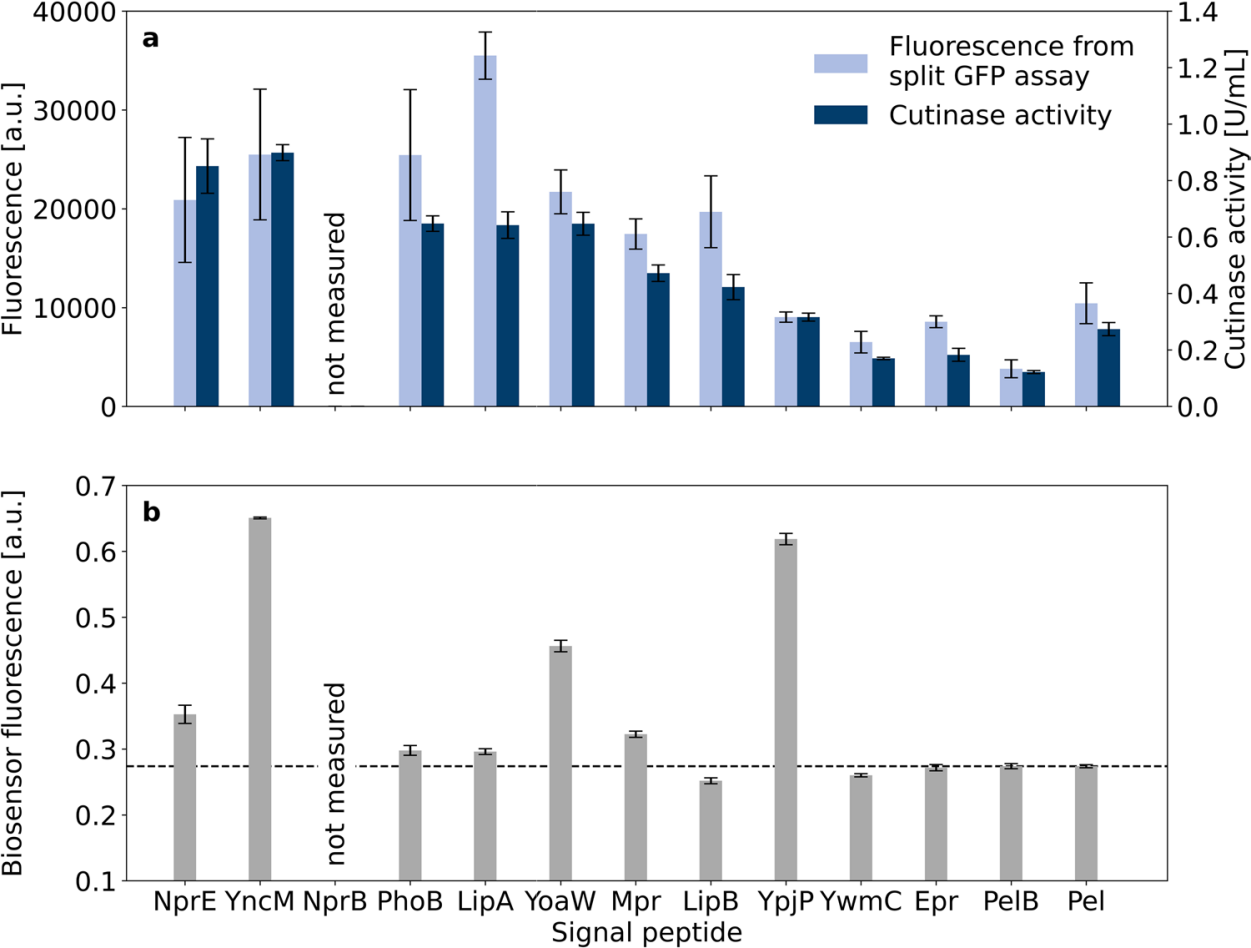
Secretion performance and secretion stress associated with cutinase-GFP11 secretion using different signal peptides in combination with an 8 nt ribosome binding site spacer. Secreted cutinase-GFP11 from *C.* *glutamicum* K9 pCMEx8-[SP]-Cutinase was detected via split GFP assay and cutinase activity measurements that were performed manually (**a**). At the end of the cultivation, the cell-specific eYFP fluorescence was determined of two independent clones and the average is shown with standard deviation (**b**). The dashed line indicates the normal background fluorescence associated with secretion stress in the absence of cutinase expression

The secretion performance of cutinase-GFP11 mediated by NprE in dependence of the spacer length in the biosensor strain showed trends similar to those observed in the wild type strain (compare Fig. 6 to Fig. 3c). However, absolute values with the *C. glutamicum* K9 biosensor strain were different due to differences in cultivation conditions, such as less glucose and 20 h of target gene expression as well as in assay conduction, such as a reduced cutinase assay volume and microplate reader settings. Importantly, the biosensor fluorescence output at the end of the cultivation correlated with the secretion performance. High absolute cutinase-GFP11 secretion was accompanied by high biosensor fluorescence, indicating increased secretion stress. Herein, the secretion stress reached a plateau for ribosome binding site spacers with 6–12 nt length (Fig. 6b), which largely coincided with the secretion performance plateau (Fig. 6a). In earlier work, it was demonstrated that biosensor cells which carry the empty vector typically exhibit a biosensor fluorescence of ~0.27 a.u. at the end of the cultivation, which represents the normal background secretion stress in the absence of cutinase expression (Bakkes et al. 2021). Therefore, this value was set as a threshold for the biosensor output (Fig. 6b, dashed line). With pCMEx4-NprE-Cutinase (Fig. 6b) and for instance pCMEx8-Pel-Cutinase (Fig. 7b), the biosensor fluorescence did not exceed 0.27 a.u., which indicates that the corresponding low-level cutinase secretion with typically less than 0.4 U/mL does not impose significant secretion stress to the cells.

In addition, the impact of different signal peptides on the cutinase secretion and the accompanying secretion stress was assessed. For this purpose, the indicated constructs all carrying an 8 nt ribosome binding site spacer were transferred to the *C.* *glutamicum* K9 biosensor strain (Fig. 7a). Importantly, the secretion performance of the different signal peptides in the biosensor strain showed trends similar to those observed in the wild-type strain (compare Fig. 7a and Fig. 5). Interestingly, also in the biosensor strain, a disproportionally high split GFP fluorescence in comparison to the cutinase activity is noted in case of the LipA and LipB signal peptides (Fig. 7a). Strikingly, cutinase export via either of these signal peptides did not lead to a substantial increase in secretion-associated stress, as evidenced by the low biosensor fluorescence output. On the other hand, the highest biosensor response was observed for cutinase-GFP11 secretion mediated by the signal peptides YncM and YpjP. Expression of the respective constructs in the wild-type strain already resulted in a slightly impaired growth phenotype, giving rise to the conclusion that the cutinase export mediated with signal peptides YncM and YpjP is accompanied by considerable secretion stress, showing also an impact on growth (Fig. 7b and Fig. 5).

## Discussion

This study highlights the benefits of laboratory automation for optimization of strain construction, protein secretion, and performance evaluation. Besides multi-purpose high-performance laboratory automation, the targeted application of low cost robotics can push the throughput far beyond the level of manual procedures. As proposed by Tenhaef et al. (2021), the Opentrons OT-2 system with additional Thermocycler and Magnetic Module is suitable for plasmid construction, which can be modularized and consecutively processed. The user can profit from the simple operation and customizable open source software for tailor-made plasmid assembly and strain construction workflows. However, the system would not be sufficient to handle more complex microbial cultivation and screening processes which require more robotic functionality and precisely timed liquid handling manipulations. In such case, more advanced liquid handling laboratory robotics is required, such as the Tecan Freedom EVO^®^ 200 with integrated BioLector^®^ Pro, centrifuge, and microplate reader. This system allows to execute more complex protocols and workflows with autonomous decision-making during the process (Jansen et al. 2019; Osthege et al. 2022b), adding a further level of complexity.

We showed that plasmid generation of 96 samples could be accelerated using automation to 55% of the manual process with significantly reduced hands-on time. Time-saving benefits were found in the transformation, plasmid purification, and test digestion for quality control, but not in the Golden Gate assembly which is performed in the thermocycler already running automatically. However, such script programming and workflow optimization need to be justified by a correspondingly large number of samples and iterations. Justification is given by the cutinase application example presented here, and by the possibility to assemble spacer-signal peptide combinations for any given protein, thereby providing a rapid strain library prototyping for alternative target proteins besides the cutinase. In addition, the individual unit operations can also be used separately for completely different molecular biological objectives, since *E. coli* shuttle vectors and methods such as heat-shock transformation, plasmid purification, and restriction digestions are widely used and small script adaptations can be easily made.

We demonstrate that automated secretion screening including pre-cultures enables high comparability between strains due to autonomous inoculation and induction events for individual cultures triggered by online biomass monitoring. This is particularly evident by the comparable duration of the main cultures, even though the lag-phases of the pre-cultures varied substantially. This is a great step towards standardization of experimental procedures providing improved reliability and reproducibility of cultivation data. In addition, the manual work is minimized and no operator supervision is required after providing cryo cultures and media on the robotic deck. One disadvantage, however, is the reduced throughput as only 48 cultivation wells are available. Here, with integrated pre-culture and main cultivation performed in triplicates, the throughput is reduced to 12 strains. Nevertheless, replicate data of the main cultures were very comparable in terms of growth phenotype and activity of secreted cutinase, paving the way to modify the workflow to run pre- and main cultures in unicates. Thus, up to 24 different strains could be tested in one run.

Results obtained from automated screening with different ribosome binding site spacer lengths for cutinase-GFP11 secretion using the *B.* *subtilis* signal peptides NprE, Pel, Bsn, and Epr in *C.* *glutamicum* revealed that in the range of the 4–12 nt spacers, the use of at least 8 nt is advised and only poor secretion was observed with the shortest spacer lengths of 4–5 nt. While shorter than 8 nt ribosome binding site spacers led to substantial performance loss, the results with 8–12 nt spacers were comparable. These results indicate that the transcriptional machinery is more sensitive to shorter than to longer ribosome binding site spacers. Similar to what was observed in *B. subtilis* secretion studies in which identical combinations of ribosome binding site spacer, signal peptide, and target protein were used (Volkenborn et al. 2020), we also observed poor cutinase secretion in *C.* *glutamicum* for short spacer lengths of 4–5 nt. These results are also consistent with reports on intracellular ornithine transcarbamoylase production in *C.* *glutamicum*. Herein, enzyme activities were decreased by reducing the ribosome binding site spacer from 8 to 5 nt (Schneider et al. 2012). With respect to longer spacer sequences, they could lead to suboptimal translation initiation, but at the same time perhaps relieve bottlenecks in other steps of the secretion process. This may result in more secreted proteins being correctly folded, which could explain the secretion performance plateau for spacers of 8–12 nt (Volkenborn et al. 2020).

Since there was no generally more suitable spacer length for the signal peptides tested for cutinase-GFP11 secretion, an automated signal peptide screening was done with the 8 nt spacer. As expected from previous studies (e.g., Hemmerich et al. 2016), secretion varies according to the signal peptide used, with NprE being the best signal peptide for cutinase secretion in *C.* *glutamicum*. In general, a good agreement was found between the cutinase activity and the split GFP assay data for most of the tested signal peptides. For LipA and LipB, however, the split GFP fluorescence was much higher than expected from the cutinase activity data. A possible explanation is that some of the secreted cutinase molecules were incompletely folded, misfolded, or partially degraded and thus enzymatically inactive, whereas the GFP11-tag is still accessible and allows for holo-GFP assembly. Misfolding and accumulation of incorrectly folded proteins upon protein export causes stress to the cells. However, when using the *C. glutamicum* K9 biosensor strain, which measures the stress associated with Sec-dependent protein translocation across the cytoplasmic membrane by fluorescence (Jurischka et al. 2020), no evidence was found that cutinase secretion via the signal peptides LipA and LipB leads to substantially increased secretion stress. It is important to note that the secretion biosensor responds to the extent of stress caused by the accumulation of incorrectly folded target protein at the *trans*-side of the cytoplasmic membrane upon Sec-dependent protein translocation, but not to the biologically active secreted target protein. Nevertheless, a general dose-dependent effect was observed between the secretion performance of an individual signal peptide and the secretion stress in dependence of the spacer length, similar to the dependence on IPTG concentrations (Jurischka et al. 2020). However, when comparing a large set of different signal peptides, it must be considered that the signal peptides may have distinct influences on the number and quality of exported cutinase molecules, e.g., by altering the synthesis, export efficiency, processing, or folding of the target protein (Freudl 2018). Because the biosensor responses for YncM- and YpjP-mediated cutinase secretion were similarly high, whereas the secretion performance with YpjP was threefold lower, the signal peptide YpjP seems to have a stronger negative effect on the cutinase quality. For both signal peptides, there appears to be a correlation to impaired growth. Taken together, the data suggest that signal peptide-specific effects may occur at later stages of Sec-dependent secretion, upon signal peptide removal or possibly even upon protein folding. Our results indicate that the biosensor strain has its limitations in applications where the screening of a large number of signal peptides that are structurally very distinct is desired.

Since the same best 5 signal peptides for cutinase-GFP11 secretion were identified with both the split GFP and the cutinase activity assay, the easily automatable split GFP assay proved to be a valuable tool for rapid strain pre-selection exhibiting high cutinase secretion. Moreover, the automated split GFP assay would be particularly useful for the detection of target proteins for which no suitable activity assay is available or in cases where the assay is strongly error-prone. For this, it must first be generally verified by other methods such as Western blots that the GFP11-tag bound to the target protein is accessible. After a pre-selection of strains, false positive hits could subsequently be discarded by manual or more elaborate methods.

## Supplementary Information

Below is the link to the electronic supplementary material.

Supplementary file1 (PDF 1247 kb)

## Data Availability

All data used for this study is provided in the article or the Supplementary Information.

## Code availability

Protocols for automated molecular biology workflows and packages for automated screening are provided in in the JuBiotech Git repository.
