# Supplementary material for: Accelerated strain construction and characterization of C. glutamicum protein secretion by laboratory automation
Source: Appl Microbiol Biotechnol. 2022 Jun 27;106(12):4481–97. doi: 10.1007/s00253-022-12017-7 (PMC9259529; doi:10.1007/s00253-022-12017-7)
Supplement: Supplementary file 1 — Supplementary file1 (PDF 1247 kb) [file 253_2022_12017_MOESM1_ESM.pdf]

# Supplementary Information

Applied Microbiology and Biotechnology

**Accelerated strain construction and characterization of *C. glutamicum* protein secretion by laboratory automation**

Carolin Müller[1, 2], Patrick J. Bakkes[1], Patrick Lenz[3], Vera Waffenschmidt[1], Laura M. Helleckes[1, 2], Karl-Erich Jaeger[1, 3], Wolfgang Wiechert [1, 4], Andreas Knapp[3, 5], Roland Freudl[1], Marco Oldiges[1, 2*]

[1] Institute of Bio- and Geosciences IBG-1: Biotechnology, Forschungszentrum Jülich GmbH, 52425 Jülich, Germany
[2] Institute of Biotechnology, RWTH Aachen University, 52062 Aachen, Germany
[3] Institute of Molecular Enzyme Technology, Heinrich Heine University Düsseldorf, Forschungszentrum Jülich, 52425 Jülich, Germany
[4] Computational Systems Biotechnology (AVT.CSB), RWTH Aachen University, 52062 Aachen, Germany
[5] Present address: Castrol Germany GmbH, 41179 Mönchengladbach, Germany
* Correspondence: m.oldiges@fz-juelich.de

**Table S1** Oligonucleotide sequences used in this study

| Oligonucleotide | 5' → 3' sequence |
|---|---|
| *Amplification of nprE-cut11 for construction of pBS-Xnt-SPNprE-Cut11:* | |
| fw-NdeI-SPNprE | gcacatatgggtttaggtaagaaattg |
| rev-GFP11-XbaI | atctctagattatgtgatgccagcagcg |
| | |
| *Circular polymerase extension cloning for construction of pCMEx12:* | |
| CPEC-Backbone_for | acgaaaggctcagtcgaaagactgg |
| CPEC-Backbone_rev | ctcatgttatatcccgccgttaacc |
| CPEC-Insert_for | ggttaacggcgggatataacatgag |
| CPEC-Insert_rev | ccagtctttcgactgagcctttcgt |
| | |
| *Exchange of spacer sequence for construction of pCMEx[4-11]:* | |
| 4nt_Spacer_for | gagaagacaggagaca |
| 4nt_Spacer_rev | tatgtctcctgtcttctctgca |
| 5nt_Spacer_for | gagaagacaggagaaca |
| 5nt_Spacer_rev | tatgttctcctgtcttctctgca |
| 6nt_Spacer_for | gagaagacaggagaaaca |
| 6nt_Spacer_rev | tatgtttctcctgtcttctctgca |
| 7nt_Spacer_for | gagaagacaggagaaaaca |
| 7nt_Spacer_rev | tatgttttctcctgtcttctctgca |
| 8nt_Spacer_for | gagaagacaggagaaaaaca |
| 8nt_Spacer_rev | tatgtttttctcctgtcttctctgca |
| 9nt_Spacer_for | gagaagacaggagaaaaaaca |
| 9nt_Spacer_rev | tatgttttttctcctgtcttctctgca |
| 10nt_Spacer_for | gagaagacaggagaaaaaaaca |
| 10nt_Spacer_rev | tatgtttttttctcctgtcttctctgca |
| 11nt_Spacer_for | gagaagacaggagaaaaaaaaca |
| 11nt_Spacer_rev | tatgttttttttctcctgtcttctctgca |

*Exchange of signal peptide sequence for construction of pCMEx[4-12]-[SP]:*

| | |
|---|---|
| Bsn_for | tatgaccaagaaggcatggttcctgccactggtgtgcgtgctgctgatctccggctggctggcaccagcagcatccgcatccgcag |
| Bsn_rev | aattctgcggatgcggatgctgctggtgccagccagccggagatcagcagcacgcacaccagtggcaggaaccatgccttcttggtca |
| Epr_for | tatgaagaacatgtcctgcaagctggtggtgtccgtgaccctgttcttctccttcctgaccatcggcccactggcacacgcag |
| Epr_rev | aattctgcgtgtgccagtgggccgatggtcaggaaggagaagaacagggtcacggacaccaccagcttgcaggacatgttcttca |
| LipA_for | tatgaagttcgtgaagcgccgcatcatcgcactggtgaccatcctgatgctgtccgtgacctccctgttcgcactgcagccatccgcaaaggcag |
| LipA_rev | aattctgcctttgcggatggctgcagtgcgaacagggaggtcacggacagcatcaggatggtcaccagtgcgatgatgcggcgcttcacgaacttca |
| LipB_for | tatgaagaaggtgctgatggcattcatcatctgcctgtccctgatcctgtccgtgctggcagcaccaccatccggcgcaaaggcag |
| LipB_rev | aattctgcctttgcgccggatggtggtgctgccagcacggacaggatcagggacaggcagatgatgaatgccatcagcaccttcttca |
| Mpr_for | tatgaagctggtgccacgcttccgcaagcagtggttcgcatacctgaccgtgctgtgcctggcactggcagcagcagtgtccttcggcgtgccagcaaaggcag |
| Mpr_rev | aattctgcctttgctggcacgccgaaggacactgctgctgccagtgccaggcacagcacggtcaggtatgcgaaccactgcttgcggaagcgtggcaccagcttca |
| NprB_for | tatgcgcaacctgaccaagacctccctgctgctggcaggcctgtgcaccgcagcacagatggtgttcgtgacccacgcatccgcag |
| NprB_rev | aattctgcggatgcgtgggtcacgaacaccatctgtgctgcggtgcacaggcctgccagcagcagggaggtcttggtcaggttgcgca |
| NprE_for | tatgggtttaggtaagaaattgtctgttgctgtcgctgcttcgtttatgagtttatcaatcagcctgccaggtgttcaggctg |
| NprE_rev | aattcagcctgaacacctggcaggctgattgataaactcataaacgaagcagcgacagcaacagacaatttcttacctaaaccca |
| Pel_for | tatgaaaaaagtgatgttagctacggctttgtttttaggattgactccagctggcgcgaacgcag |
| Pel_rev | aattctgcgttcgcgccagctggagtcaatcctaaaaacaaagccgtagctaacatcactttttca |
| PelB_for | tatgaagcgcctgtgcctgtggttcaccgtgttctccctgttcctggtgctgctgccaggcaaggcactgggcg |
| PelB_rev | aattcgcccagtgccttgcctggcagcagcaccaggaacagggagaacacggtgaaccacaggcacaggcgcttca |
| PhoB_for | tatgaagaagttcccaaagaagctgctgccaatcgcagtgctgtcctccatcgcattctcctccctggcatccggctccgtgccagaagcatccgcag |
| PhoB_rev | aattctgcggatgcttctggcacggagccggatgccagggaggagaatgcgatggaggacagcactgcgattggcagcagcttctttgggaacttcttca |
| YoaW_for | tatgaagaagatgctgatgctggcattcaccttcctgctggcactgaccatccacgtgggcgaagcatccgcag |
| YoaW_rev | aattctgcggatgcttcgcccacgtggatggtcagtgccagcaggaaggtgaatgccagcatcagcatcttcttca |
| YpjP_for | tatgaagctgtggatgcgcaagaccctggtggtgctgttcaccatcgtgaccttcggcctggtgtccccaccagcagcactgatggcag |
| YpjP_rev | aattctgccatcagtgctgctggtggggacaccaggccgaaggtcacgatggtgaacagcaccaccagggtcttgcgcatccacagcttca |
| YwmC_for | tatgaagaagcgcttctccctgatcatgatgaccggcctgctgttcggcctgacctccccagcattcgcag |
| YwmC_rev | aattctgcgaatgctggggaggtcaggccgaacagcaggccggtcatcatgatcagggagaagcgcttcttca |

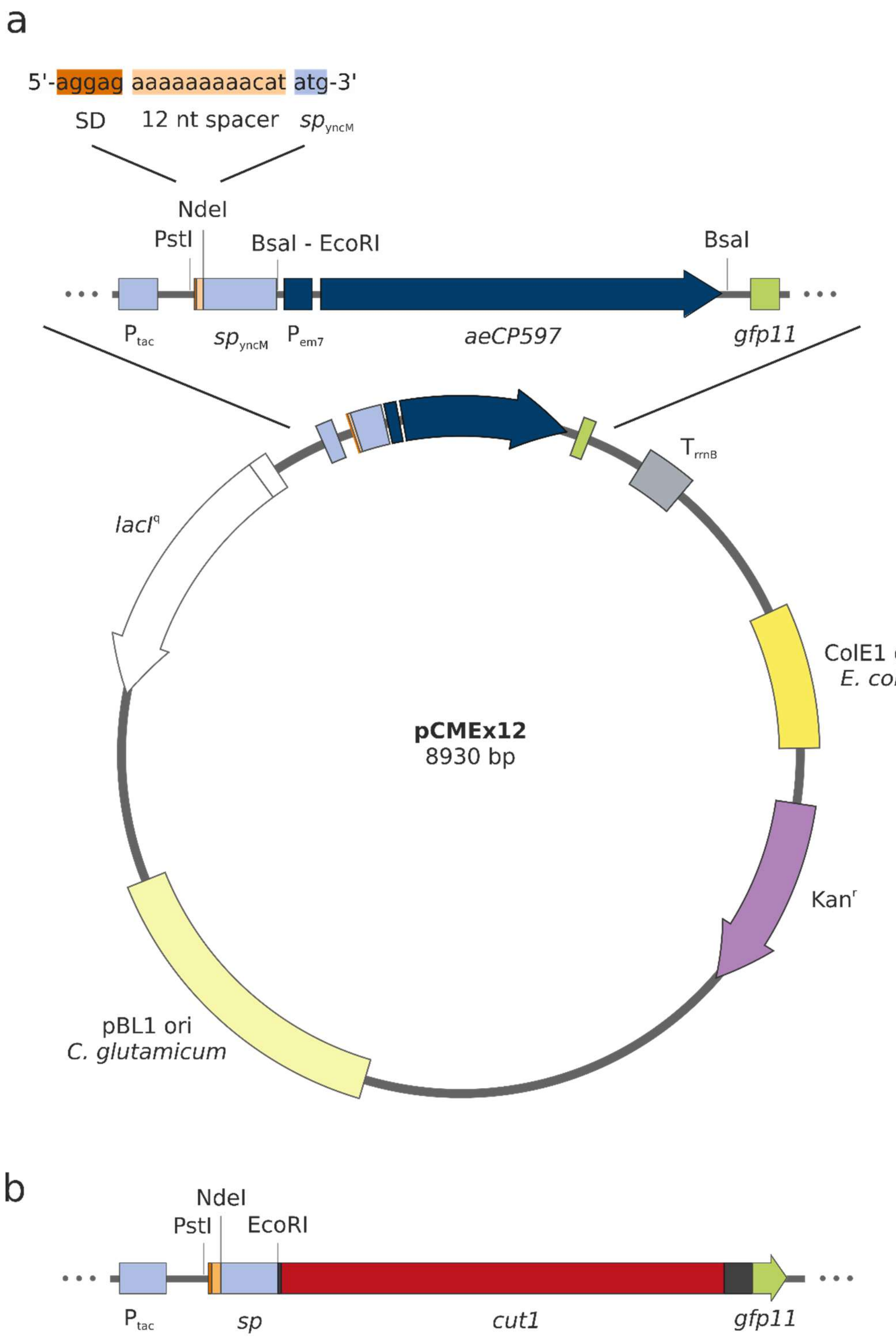


**Fig. S1** Sequence features of pCMEx-based plasmids. Plasmid map of pCMEx12 (a). The expression cassette contains a tac promoter, a 12 nt ribosome binding site spacer between the Shine-Dalgarno sequence (SD) and the *B. subtilis* signal peptide sequence from YncM, the GFP11-tag sequence and a rrnB terminator. The *Actinia equina blue chromoprotein aeCP597* under the control of the em7 promoter between the signal peptide sequence and the GFP11-tag sequence is constitutively expressed. After Golden Gate assembly, the cutinase gene *cut1* is in frame with the signal peptide and GFP11-tag sequence under the control of the IPTG-inducible tac promoter (b). Two amino acids (Glu, Phe) connect the signal peptide with the target protein after translation. The GFP11-tag is attached to the target protein *via* a polypeptide linker consisting of 14 amino acids. Partially created with SnapGene® software (from Insightful Science; available at snapgene.com)

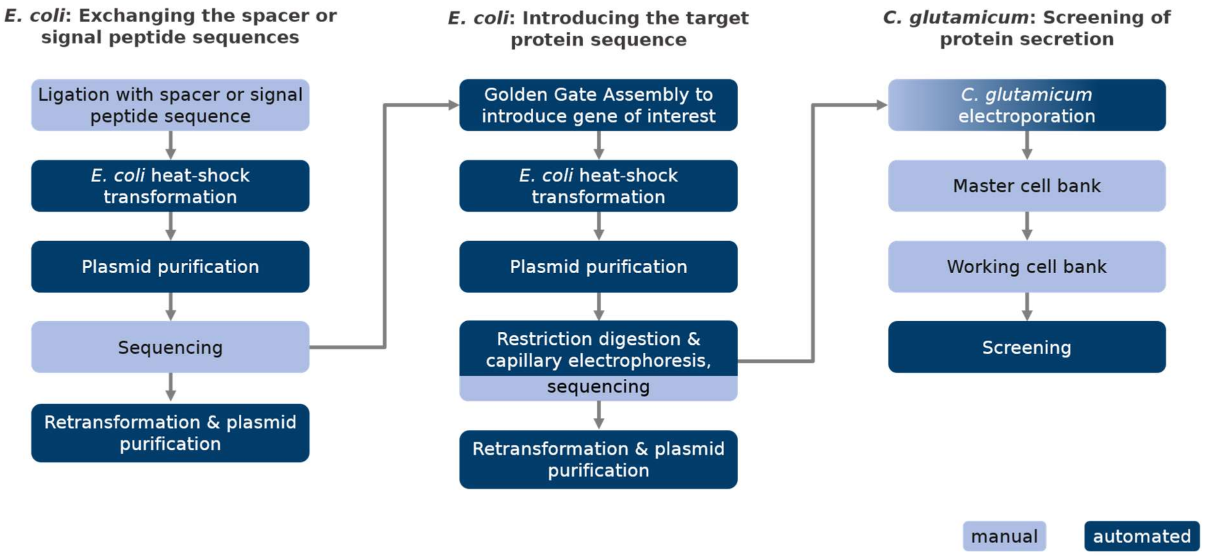


**Fig. S2** Workflow from exchanging plasmid sequences to automated secretion screening. Automated steps are highlighted in dark blue and manual steps in light blue. Molecular cloning is done using *E. coli* and steps can be divided into the exchange of ribosome binding site spacer or signal peptide sequences by cassette mutagenesis and introduction of the target gene by Golden Gate assembly. Only sequence verified expression plasmids are introduced in *C. glutamicum* by electroporation, which cannot be automated but parallelized by using a 96-well electroporation device. Only the screening is automated using a Tecan Freedom EVO® 200 with integrated BioLector® Pro, centrifuge and microplate reader. All other steps were automated using the Opentrons OT-2 system with additional Thermocycler and Magnetic Module

**Table S2** Golden Gate assembly. Categories are M: manual step, I: manually timed incubation and A: automated step

| Step | Automated [min] | | | Manual [min] | | |
|---|---|---|---|---|---|---|
| | M | I | A | M | I | A |
| Prepare robotic | 10 | - | - | - | - | - |
| Prepare reaction mix | 5 | - | - | 5 | - | - |
| Add pre-diluted DNA to plate | - | - | 5 | 5 | - | - |
| Mix reaction mix with DNA | - | - | 10 | 10 | - | - |
| Thermocycler protocol | - | - | 210 | - | - | 210 |
| Finish and take plate | 5 | - | - | 5 | - | - |
| Sum [min] | 20 | 0 | 225 | 25 | 0 | 210 |
| Total time [min] | | 245 | | | 235 | |

**Table S3** Heat-shock transformation. Categories are M: manual step, I: manually timed incubation and A: automated step

| Step | Automated [min] | | | Manual [min] (12 samples) | | | Manual (2x 12 samples, nested) | | |
|---|---|---|---|---|---|---|---|---|---|
| | M | I | A | M | I | A | M | I | A |
| Prepare robotic | 20 | - | - | - | - | - | - | - | - |
| Mixing cells and DNA | - | - | 10 | 10 | - | - | 20 | - | - |
| Incubation on ice | - | - | 30 | - | 20 | - | - | 40 | - |
| Heat-shock and cooling | - | - | 3 | 5 | - | - | 10 | - | - |
| Regeneration | - | - | 70 | 5 | 60 | - | 10 | 40[1] | - |
| Streaking or plating out | 5 | 10 | 7 | 20 | - | - | 40 | - | - |
| Sum [min] | 25 | 10 | 120 | 40 | 80 | 0 | 80 | 80 | 0 |
| Total time [min] | | 155 | | | 120 | | | 160 | |

[1] 2x 60 min regeneration in total, but only 40 min of it are considered for calculation, because in the remaining 80 min other steps can be done in parallel

**Table S4** Plasmid preparation. Categories are M: manual step, I: manually timed incubation and A: automated step

| Step automated | Automated [min] | | | Manual [min] (24 samples) | | | Step manual |
|---|---|---|---|---|---|---|---|
| | M | I | A | M | I | A | |
| Prepare robotic, cell harvest | 20 | - | - | 25 | - | - | Cell harvest |
| Automated protocol part I | - | - | 70 | 15 | - | - | Resuspend cells |
| Tip refill exchange of plates | 5 | - | - | 10 | - | - | Precipitation |
| Automated protocol part II | - | - | 90 | 10 | - | - | Pellet cell debris |
| Drying samples | - | 35 | - | 25 | - | - | Washing and elution |
| Automated protocol part III | - | - | 30 | 5 | - | - | Label and store tubes |
| Seal and store plate | 5 | - | - | - | - | - | - |
| Sum [min] | 30 | 35 | 190 | 90 | 0 | 0 | Sum [min] |
| Total time [min] | | 255 | | | 90 | | Total time [min] |

**Table S5** Test digestion. Categories are M: manual step, I: manually timed incubation and A: automated step

| Step | Automated [min] | | | Manual [min] | | |
|---|---|---|---|---|---|---|
| | M | I | A | M | I | A |
| Prepare robotic | 10 | - | - | - | - | - |
| Prepare reaction mix | 10 | - | - | 10 | - | - |
| Mix DNA and reaction mix | - | - | 8 | 25 | - | - |
| Digestion and heat inactivation | - | - | 92 | - | 80 | - |
| Dilution of samples | 5 | - | 15 | 15 | - | - |
| Sum [min] | 25 | 0 | 115 | 50 | 80 | 0 |
| Total time [min] | | 140 | | | 130 | |

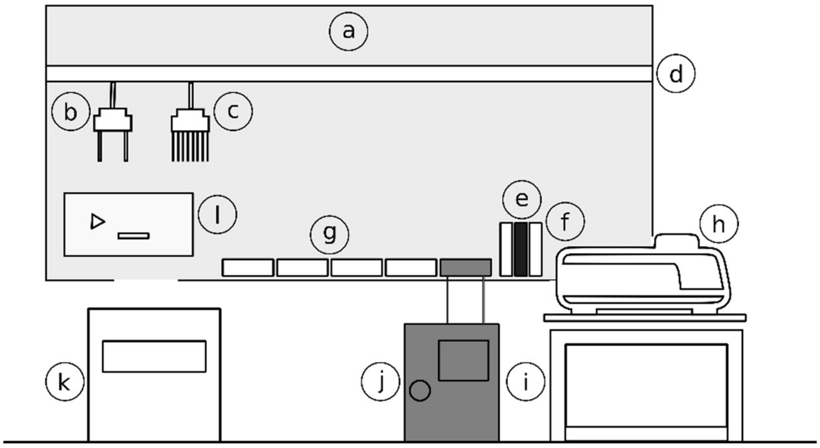


**Fig. S3** Schematic of the robotic screening platform used in this study. The Tecan Freedom EVO® with a 200 base unit is equipped with (a) a sterile hood, (b) a unit for moving the (c) liquid handler and (d) robotic manipulator, (e) a washing station, (f) trough carriers, (g) plate carriers, (h) a BioLector® Pro with (i) control unit, (j) a cooling system and cooling carrier for plates, (k) a plate centrifuge and (l) microplate reader

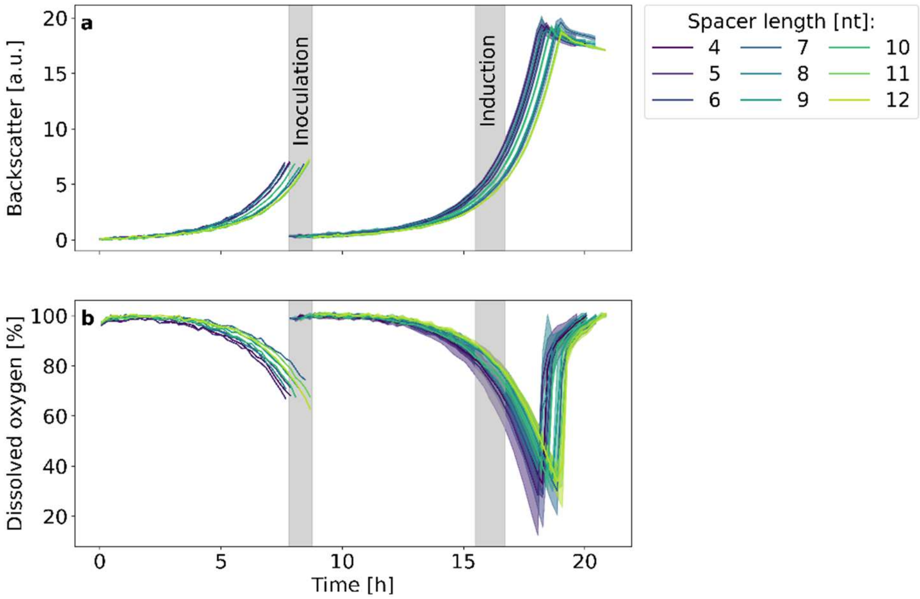


**Fig. S4** Pel-Cutinase-GFP11 secretion with ribosome binding site spacer lengths from 4–12 nt. Backscatter (a) and dissolved oxygen (b) were measured during cultivation of *C. glutamicum* pCMEx[4-12]-Pel-Cutinase. Pre-cultures inoculated from cryo cultures were used to inoculate three main cultures that are shown as mean with standard deviation in confidence tubes. Inoculation and induction of main cultures with IPTG to a final concentration of 200 µM were each triggered by a device-dependent backscatter signal in the exponential phase. Cells were harvested 4 h after induction with main culture durations of 12–12.2 h

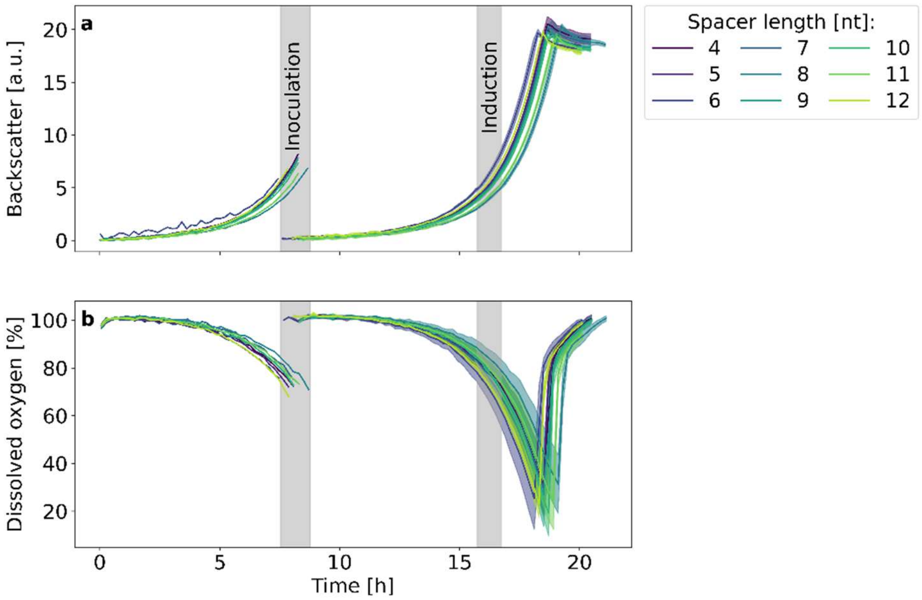


**Fig. S5** Epr-Cutinase-GFP11 secretion with ribosome binding site spacer lengths from 4–12 nt. Backscatter (a) and dissolved oxygen (b) were measured during cultivation of *C. glutamicum* pCMEx[4-12]-Epr-Cutinase. Pre-cultures inoculated from cryo cultures were used to inoculate three main cultures that are shown as mean with standard deviation in confidence tubes. Inoculation and induction of main cultures with IPTG to a final concentration of 200 µM were each triggered by a device-dependent backscatter signal in the exponential phase. Cells were harvested 4 h after induction with main culture durations of 12–12.4 h

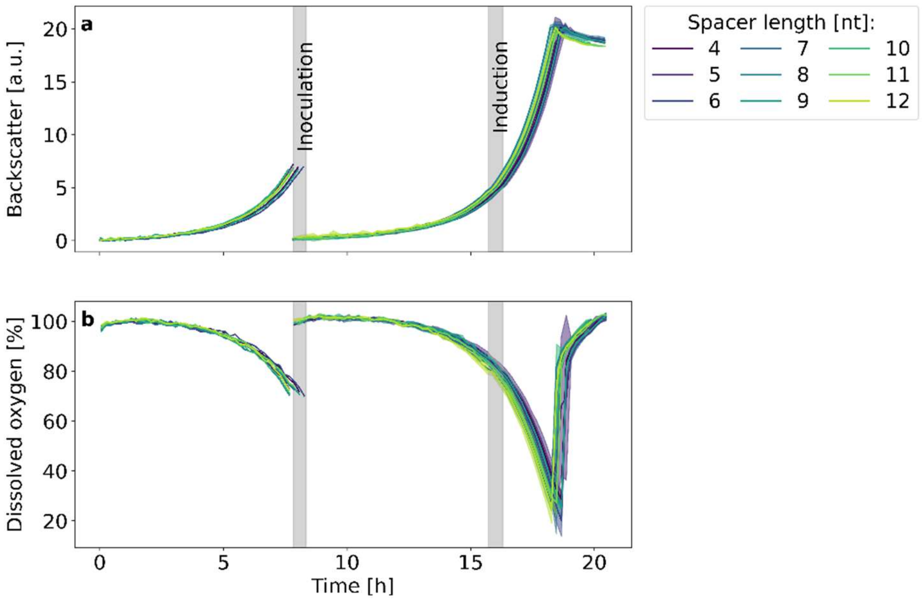


**Fig. S6** Bsn-Cutinase-GFP11 secretion with ribosome binding site spacer lengths from 4–12 nt. Backscatter (a) and dissolved oxygen (b) were measured during cultivation of *C. glutamicum* pCMEx[4-12]-Bsn-Cutinase. Pre-cultures inoculated from cryo cultures were used to inoculate three main cultures that are shown as mean with standard deviation in confidence tubes. Inoculation and induction of main cultures with IPTG to a final concentration of 200 µM were each triggered by a device-dependent backscatter signal in the exponential phase. Cells were harvested 4 h after induction with main culture durations of 11,9–12.4 h
